# Solvent-Free Design of Biobased Non-isocyanate Polyurethanes with Ferroelectric Properties

**DOI:** 10.1021/acssuschemeng.1c05380

**Published:** 2021-10-27

**Authors:** Valentina Sessini, Cuong Nguyen Thai, Harvey Amorín, Ricardo Jiménez, Cédric Samuel, Sylvain Caillol, Jérôme Cornil, Sébastien Hoyas, Sophie Barrau, Philippe Dubois, Philippe Leclère, Jean-Marie Raquez

**Affiliations:** † Laboratory of Polymeric and Composite Materials, Center of Innovation and Research in Materials and Polymers (CIRMAP), University of Mons—UMONS, Place du Parc 23, 7000 Mons, Belgium; ‡ Laboratory for Chemistry of Novel Materials (SCMN), Center of Innovation and Research in Materials and Polymers (CIRMAP), University of Mons—UMONS, Place du Parc 23, 7000 Mons, Belgium; § Université de Lille, CNRS, INRAE, Centrale Lille, UMR 8207—UMET—Unité Matériaux et Transformations, F-59000 Lille, France; ∥ Instituto de Ciencia de Materiales de Madrid (ICMM), CSIC, Cantoblanco, 28049 Madrid, Spain; ⊥ IMT Lille Douai, Institut Mines-Télécom, Univ. Lille, Centre for Materials and Processes, F-59000 Lille, France; # ICGM, Université de Montpellier, CNRS, ENSCM, UMR 5253, Place Eugène Bataillon CC 1700-Bâtiment 17, 34095 Montpellier cedex 5, France; ¶ Organic Synthesis & Mass Spectrometry Laboratory, Interdisciplinary Center for Mass Spectrometry (CISMa), Center of Innovation and Research in Materials and Polymers (CIRMAP), University of Mons—UMONS, Place du Parc 23, 7000 Mons, Belgium

**Keywords:** non-isocyanate polyurethanes, solvent-free methods, reactive extrusion, ferroelectricity, biobased polymers

## Abstract

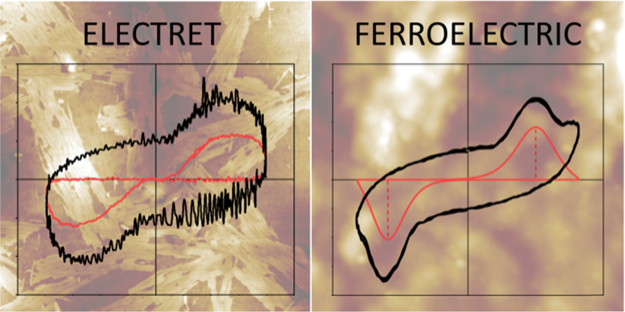

Increasing energy autonomy and lowering dependence on lithium-based batteries are more and more appealing to meet our current and future needs of energy-demanding applications such as data acquisition, storage, and communication. In this respect, energy harvesting solutions from ambient sources represent a relevant solution by unravelling these challenges and giving access to an unlimited source of portable/renewable energy. Despite more than five decades of intensive study, most of these energy harvesting solutions are exclusively designed from ferroelectric ceramics such as Pb(Zr,Ti)O_3_ and/or ferroelectric polymers such as polyvinylidene fluoride and its related copolymers, but the large implementation of these piezoelectric materials into these technologies is environmentally problematic, related with elevated toxicity and poor recyclability. In this work, we reveal that fully biobased non-isocyanate polyurethane-based materials could afford a sustainable platform to produce piezoelectric materials of high interest. Interestingly, these non-isocyanate polyurethanes (NIPUs) with ferroelectric properties could be successfully synthesized using a solvent-free reactive extrusion process on the basis of an aminolysis reaction between resorcinol bis-carbonate and different diamine extension agents. Structure–property relationships were established, indicating that the ferroelectric behavior of these NIPUs depends on the nanophase separation inside these materials. These promising results indicate a significant potential for fulfilling the requirements of basic connected sensors equipped with low-power communication technologies.

## Abstract 2

Improved compatibility results in better mechanical coupling between soft and hard phases of fully biobased NIPUs produced by a solvent-free process.

## Introduction

Our last decades were being driven for the continuous search of more sustainable alternatives to reduce the overconsumption of fossil fuels, relating to the global warming. Renewable energy sources such as wind and solar energies can represent a relevant option to overcome this issue but are not sufficient to fully support the global energy demand in the upcoming decades. Considering these factors, academic and industrial researchers are now pushing to develop advanced energy harvesting technologies from wasted energy sources as an alternative to the aforementioned issues.^1^

In this context, piezoelectric materials are expected to emerge as one of the most important functional materials for the energy harvesting sector.^2,3^ Piezoelectric materials are able to convert the mechanical energy to electrical signals, named the direct piezoelectric effect also known as generator or transducer effects, as well as the opposite, called the converse piezoelectric effect also known as motor/actuator effects.^1,4^ Interestingly, the energy harvesting with such materials represents the most suited solution to power electronic devices of low voltage such as wireless sensors, portable devices, and medical implants, particularly in Internet of things (IoT).^4^ They can even represent a unique solution in many applications like sensors installed in remote locations or inside the human body in which external intervention to replace these devices after their service remains difficult or even impossible.^5^ In this respect, piezoelectric energy harvesters provide the best promises because they can generate elevated electrical energy density that can be readily stored in any battery-related system. In addition, the high flexibility of some piezoelectric materials allows them to be readily downsized into miniaturized devices.^6^ In general, piezoelectric materials can be divided into four main categories depending upon their microstructural characteristics: ceramics, single crystals, polymers, and composites.^7^

Among them and related with economic and environmental issues,^8^ piezoelectric polymers afford several advantages because they are easily processable on a large scale and can be readily designed in different shapes and useful objects like thin films. Moreover, they are flexible, lightweight, and show interesting mechanical properties when compared to extremely fragile ceramic materials. Even though the piezoelectric performance of polymers remains lower than that of ceramics, they have much higher piezoelectric voltage coefficients, being very interesting in the case of sensing applications.^5,9^

The piezoelectric effect in these polymers is mainly due to their specific (macro)molecular structure that can be achieved in amorphous and semi-crystalline materials.^10^ Even if they are characterized by different mechanisms, giving rise to piezoelectricity, four critical features must be present within all these piezoelectric polymers. These are (1) the presence of permanent molecular dipoles, (2) the ability to orient or align the molecular dipoles, (3) the ability to fix this dipole alignment once it is achieved, and (4) the ability of the material to undergo large strains when mechanically stressed.^11^

Semi-crystalline polymers contain a polar polymorphic crystalline phase that can be readily orientated by poling treatments, leading to the piezoelectric effect. In the case of amorphous polymers, they have to be of vitreous nature and contain within their macromolecular structure an elevated number of molecular dipoles, which might be aligned and fixed to give rise to piezoelectricity.^12^

Polyvinylidene fluoride (PDVF) and its copolymers represent the best examples of ferroelectric semi-crystalline polymers with excellent piezoelectric and pyroelectric properties,^13−16^ but they are not considered as green as their sourcing and production are both impactful.^17^ Therefore, other types of polymers have been extensively investigated in the last decade and significant efforts are more and more being focused on sustainable and environmentally friendly ferroelectric polymers. Among them, polyurethanes (PUs) represent the most versatile polymers nowadays.^2,18−21^ They are the sixth most widely used polymers being applied in many industrial fields with a variety of physical, chemical, and interesting electroactive properties. The structural and ferroelectric properties of aliphatic PUs were first reported by Tasaka et al.,^22^ and it was suggested that ferroelectricity was originated from the hydrogen-bonded crystalline regions. On the other hand, amorphous aromatic PUs have also been claimed to be ferroelectrics, whose origin was attributed to dipole motions in the hydrogen-bonded amorphous phase, that can be frozen below *T*_g_.^12,23,24^ This is even higher compared that of aliphatic PUs due to the chain stiffening with bulky phenyl groups. Despite the large number of applications of PUs, increasing concerns related to the toxicity of isocyanate monomers currently used for their synthesis, moisture sensitivity, and sustainability have stimulated the search for sustainable and alternative synthetic strategies for these materials.^25^

Non-isocyanate PUs (NIPUs) produced from cyclic carbonate aminolysis are materials with high potential to replace traditional PUs.^26,27^ Despite being promising, NIPUs suffer from some limitations even if a catalyst is used, such as conversion limitations that prevent obtaining high-molecular mass materials. Indeed, during the NIPU formation, extra hydrogen bonds are steadily created between reactive species and hydroxyl and carbamate groups, leading to an increase in the viscosity of the system and consequently limiting the reactivity and thus the final molecular weight of NIPUs.^28,29^ The production of these promising functional materials using efficient processes represents a significant challenge. Compared with traditional PUs, NIPUs exhibit improved thermal stability, higher resistance to nonpolar chemical solvents, increased adhesion and wear resistance.^25,30^ In addition, sustainability will be essential for future technologies, being the main core to harmonize our living environment with nature.^8^ In this direction, researchers and industries are directing many efforts toward the synthesis of biobased active polymers using environmentally friendly and solvent-free methods in the last decades.^31−33^ Interestingly, the main biobased cyclic bis-carbonates that are used for the production of NIPUs by aminolysis can be derived from CO_2_-sourced carbonated vegetable oils^34−36^ and more recently by the use of some biobased aliphatic, cyclic, and aromatic amines in the field.^37^

In this work, fully biobased NIPUs with ferroelectric properties have been successfully synthesized using a solvent-free process. For the best of our knowledge, ferroelectric properties of NIPUs have never been reported in the literature. In order to achieve these ferroelectric properties, the structure of the NIPUs has been finely tuned up to get a high *T*_g_ by using a biobased aromatic bis-cyclic carbonate monomer such as resorcinol bis-carbonate in the presence of two short biobased diamines, that is, putrescine (or 1,4-diaminobutane) and cadaverine (or 1,5-diaminopentane). To increase the flexibility of these NIPUs, the as-synthesized biobased diamine oligomer was also investigated as a chain extender in order to study how the molecular architecture and the flexibility can affect the final polar structure and consequently the ferroelectric behavior. To design these NIPU materials in a sustainable manner, reactive extrusion (REX) was employed, representing an attractive and sustainable solvent-free polymer processing route.^36,38−40^ The benefits of the REX process were studied, and the (nano)morphology of the NIPUs obtained was characterized deeply by atomic force microscopy (AFM).

## Results and Discussion

### Solvent-Free Production of NIPUs and Chemical Characterization

**Figure 1:**
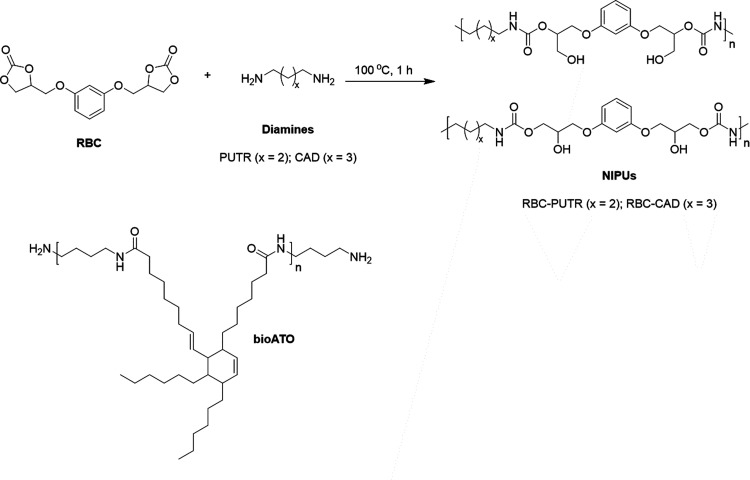
Synthesis of RBC-based NIPUs (*x* = 2 for PUTR or *x* = 3 for CAD).

NIPUs were first synthesized from resorcinol bis-cyclic carbonate (RBC) and two aliphatic diamines, respectively, cadaverine (CAD, RBC–CAD being the resulting NIPU) and putrescine (PUTR, RBC–PUTR being the resulting NIPU), as reported in Figure 1.

The reactions were performed at 100 °C for 1 h, while the screw speed was maintained constant at 100 rpm (see Supporting Information). With the aim to increase the flexibility of the NIPU structure and study how the architecture can affect its thermal, morphological, and ferroelectric properties, a NIPU based on the RBC–CAD system was synthesized in the presence of an amino-telechelic oligoamide (bioATO) as a chain extender. In fact, thanks to its oligomeric aliphatic structure and chain length, bioATO could increase the flexibility of the rigid RBC–CAD-type system originating from the aromatic ring of RBC.

Several authors have reported that reaction temperatures higher than 120 °C can lead to the undesirable formation of different side products such as ureas and oxazolidinones that can affect negatively the conversion rate and the NIPU molecular weight.^41,42^ Therefore, in our case, the polymerization temperature inside the extruder was chosen as a balance between the melting flow of the monomers and the polymer already formed and the maximum temperature to avoid these side reactions. As mentioned in the synthesis procedure, the liquid monomers were premixed before being injected into the extruder in order to facilitate their introduction. Based on this strategy, we ensured that the pressure induced by the screws is efficient enough to provide the correct flow of the material within the extruder and therefore to achieve the successful synthesis of these NIPUs.

The reaction extent was followed by monitoring uniaxial force recorded over time^39^ (Figure S1). More specifically, the uniaxial force is expected to increase with the melt viscosity as a result of the molar mass increase of NIPUs. Figure S1a shows that after 1 h, the melt viscosity of RBC–CAD was no longer increasing and even reached a plateau. In contrast, the melt viscosity of even RBC–PUTR reached higher values and the plateau of the curve was not still observed after 1 h, indicating that the polymerization was still not completed. After this reaction time, even if the reaction was not complete, RBC–PUTR was recovered back in order to avoid any possible thermal degradation. The same behavior was observed in the case of RBC–CAD-bioATO even after 90 min in which no plateau was observed, suggesting that the polymerization was almost uncompleted (Figure S1b). However, in both cases, the increase in uniaxial force was more elevated and occurred in shorter residence time, about 10 and 15 min for RBC–PUTR and RBC–CAD-bioATO, respectively. Similar results were obtained by Poussard et al.,^43^ and this behavior was justified by the reduced accessibility of amine moieties present in the end groups of the oligoamide derivatives toward the ring-opening reaction of our cyclic carbonate derivative, as ascribed to the steric hindrances commonly reported for this kind of diamine.

After extrusion, RBC–CAD and RBC–PUTR were analyzed by infrared spectroscopy [Fourier-transform infrared (FTIR)] and proton NMR techniques. In Figure S2, the FTIR spectra of the monomers and the obtained biobased NIPUs are shown. FTIR spectra confirmed the NIPU synthesis by REX. In fact, it is easy to notice that the intensity of the signal characteristic of the carbonyl group of RBC^44^ at 1782 cm^–1^ decreased for all the NIPUs processed, totally disappearing in the case of RBC–CAD and RBC–PUTR. Contrary to what was observed following the reaction extent during the REX, RBC–PUTR spectra showed a complete conversion probably because the residual amount of RBC is so low, and no change was noticed in the IR spectra. Furthermore, the band related to the urethane bond was observed at 1692 cm^–1^, demonstrating that the reaction took place and the hydroxyurethane functionality was formed. Compared with traditional PUs, this band is shifted toward lower wavenumber, indicating that these hydroxyurethane bonds are hydrogen bonded in all NIPUs.^44−46^ Moreover, it was possible to observe the presence of a broad band at 3330 cm^–1^ related to the −OH groups characteristic of NIPUs.^44−47^

The ^1^H NMR spectra of NIPUs demonstrate their successful formation, confirming the high conversion reached during REX (Figures S6 and S7). In fact, in the case of RBC–CAD and RBC–PUTR, no characteristic signal of the RBC ring was observed after 1 h extrusion, demonstrating that the carbonate conversion was over 99%. In addition, ^1^H NMR spectra show that no urea was formed during the reactive compounding of the NIPUs at 100 °C. Furthermore, no signal was observed at 5.7 ppm that could be clearly attributed to this kind of functionality.^48^ In the case of RBC–CAD-bioATO, the resulting NIPU was insoluble in current deuterated solvent, making difficult to deeply study its chemical structure by NMR analysis, including its macromolecular characterization using GPC. However, the samples RBC–CAD and RBC–PUTR result in partially soluble NIPUs due to the presence of high hydrogen bonding, not enabling an accurate determination of their molecular weight.

### Thermal Properties and Stability

Thermal properties of the synthesized NIPUs were then studied by differential scanning calorimetry (DSC) and thermogravimetric analysis (TGA). The thermal decomposition of NIPUs was assessed by TGA under nitrogen (Figure S8) while Figure S9 shows the second heating scan of DSC analysis. Table 1 summarize all thermal properties.

**Table 1:** Thermal Properties of the NIPUs Obtained by REX

	DSC			TGA	
	*T*_g_ [°C]	*T*_m_ [°C]	*T*_c_ [°C]	*T*5% [°C]	*T*_max_ [°C]
RBC	7/15			174	239/331
RBC–CAD	39			204	289/320
RBC–PUTR	50			223	275/312
bioATO	–11	94	84	364	427
RBC–CAD-bioATO	20/42	96	83	237	442

The thermal properties are affected by the diamine chain length; the longer the diamine, the lower the glass transition temperature, as it was previously demonstrated by other authors.^39,47^ From Figure S9a, NIPUs synthesized from RBC and CAD or PUTR were amorphous materials with a *T*_g_ of 39 and 50 °C, respectively. Similar results have been obtained for other NIPUs based on RBC. Schimpf et al.^30^ synthesized an amorphous NIPU based on RBC and 1,12-diaminododecane with a *T*_g_ of 35 °C. When our biosourced diamine oligoamide was used as a chain extender, a semi-crystalline NIPU was obtained, with double *T*_g_ and a *T*_m_ at 96 °C. As it is easy to notice in Figure S9b, the *T*_m_ observed was closely related to the semicrystalline character of the neat bioATO.

On the other hand, the thermal stability of the resulting NIPU has the opposite tendency, compared with the *T*_g_ results, that is, the longer the aliphatic diamine, the higher the NIPU thermal stability.^39^ The thermal stability of the NIPUs based on CAD or PUTR as diamine was considerably lower than that of RBC–CAD-bioATO, in which RBC–PUTR was the less thermally stable NIPU, as seen from the *T*_max_ values. In particular, RBC–CAD-bioATO exhibited a jump higher than 100 °C compared to the other NIPUs obtained.

Indeed, the higher thermal stability is related to the increase in the length along the adjacent hydroxyurethane bonds as previously reported.^49^ Interestingly, the thermal stability of RBC–CAD-bioATO was demonstrated being higher than that of traditional PUs that degrade at around 310 °C as reported in the literature.^50,51^ Regarding the degradation pathway, RBC–CAD and RBC–PUTR showed two *T*_max_, indicating that two degradation steps occur. The first step corresponds to the degradation of urethane links, while the second one corresponds to the degradation of the main hydrocarbon chain.^52^ In the case of RBC–CAD-bioATO, a major single degradation step was noticed probably due to the % amount of bioATO used.

### Microstructure Characterization by AFM

**Figure 2:**
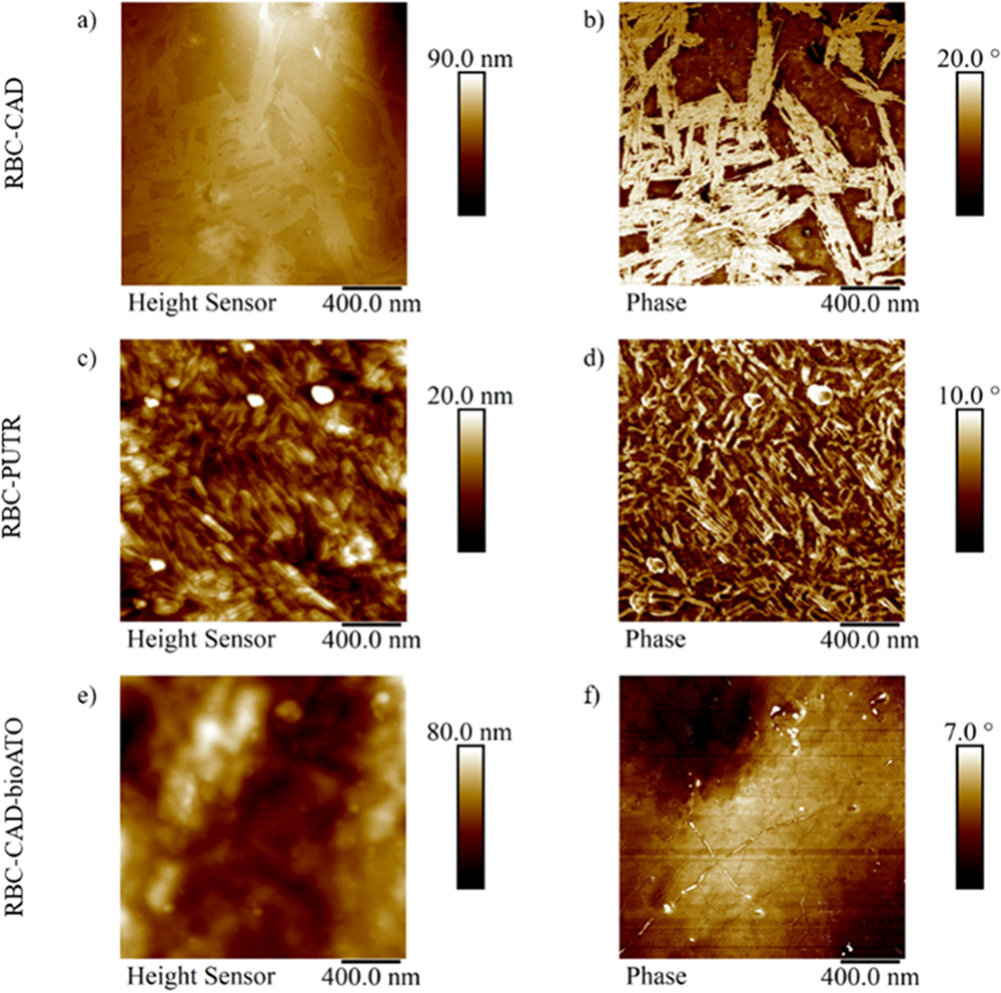
Comparison of the morphology of (a,b) RBC–CAD, (c,d) RBC–PUTR, and (e,f) RBC–CAD-bioATO, prepared by compression molding.

The microstructure of the obtained NIPUs was studied by AFM. This technique is widely accepted as a versatile method for studying microstructures of both conventional PUs and NIPUs.^53−55^ The AFM samples of the NIPUs obtained by polyaddition of RBC to PUTR or CAD were prepared by drop casting and by compression molding. For drop-cast samples, there is a significant difference in surface morphologies between RBC–CAD and RBC–PUTR in which RBC–CAD showed needle-like structures. By contrast, RBC–PUTR exhibited a nanophase-mixing surface topography as observed in Figures S10 and S11. On the other hand, samples prepared by compression molding of both RBC–CAD and RBC–PUTR showed phase-separated nanostructurations although they are totally amorphous (Figure 2a–d).

When RBC–CAD was processed by drop casting method, the mobility of the polymer chain was more increased, having more time to form a nanostructuration into longer and ordered needle (Figure S11). On the other hand, when RBC–CAD was processed by compression molding, a lamellar nanostructuration was observed (Figure 2). Nevertheless, the following AFM analysis was performed only for the compression-molded samples because they are synthesized by solvent-free method and to study the microstructure of the samples as they are used for the proposed application.

As previously reported, phase images in tapping AFM are well-suited for displaying domains of different mechanical properties inside PU-based samples.^54,56^ In our case, the contrast between the nanostructures and the matrix is apparent. Especially, for the RBC–CAD-bioATO sample, fiber-like domains, which were obscured in the height images, were revealed in phase images (Figure S12). It should be noted that the degree of nanophase separation using the oligoamide (RBC–CAD-bioATO) was less prominent with respect to the other two samples. The nanophase separation could be ascribed to the high level of interurethane hydroxy hydrogen bonding present in the hard or soft domains of NIPUs. Some previous studies of nanophase separation due to hydrogen bonding in PUs and NIPUs monitor the peak associated with urethane carbonyl, concluding that shifts at ∼1690 and ∼1720 cm^–1^ are associated with hydrogen-bonded carbonyl and free carbonyl, respectively.^35,44,57^ As shown in Figure S13a, both RBC–CAD and RBC–PUTR showed a high level of hydrogen-bonded carbonyl (∼1690 cm^–1^), whereas a high level of free carbonyl (∼1720 cm^–1^) was much more evident in the spectrum of RBC–CAD-bioATO. It should be noted that, besides the hydrogen bonds between hard domains of soft domains, hydrogen bonding can also be formed between hard and soft domains, which in turn results in phase mixing. When −O– groups in soft domains form hydrogen bonds with −NH groups in hard domains, a part of carbonyl in the hard domain will be dissociated. Therefore, the FTIR results indicated that the RBC–CAD-bioATO sample had a large portion of hard segments dissolved in soft segments, resulting in a reduction in the nanophase separation.

Moreover, when bioATO had been added as a chain extender in RBC–CAD-based NIPU, the nanostructure was increasingly well-distributed compared to that in RBC–CAD. This suggests that the compatibility between components was enhanced. It should be noted that due to the semicrystalline character of bioATO, the morphology of NIPU can be frozen in its crystalline phase, leading to a reduction in the phase-separation, as previously reported.^58^ In order to prove that the nanostructuration depends on the inter-urethane hydrogen bonding, the morphological study by AFM was assessed as a function of the increased temperature. As seen in Figure S14, the RBC–CAD nanostructure observed at 25 °C was still stable up to 35 °C. At these temperatures, the nanostructures composed of chains packed tightly together with a slight organization order. At 40 °C, the nanostructure started to change dramatically. All chains were relaxing, and the orderliness started to fade down. Furthermore, at 70 and 75 °C, the height at the middle of the image was lower than that at the edge. This can be explained by the fact that the hardness of the surface was reduced to a point that the tip could sweep the material from the center to both sides (the force was kept constant all the time). It is worth to notice that when the sample was cooled down to room temperature, the nanostructure appeared again but with different organization. Thus, we can conclude that the nanostructures are likely stable below glass transition temperature while they are significantly altered above this temperature.

**Figure 3:**
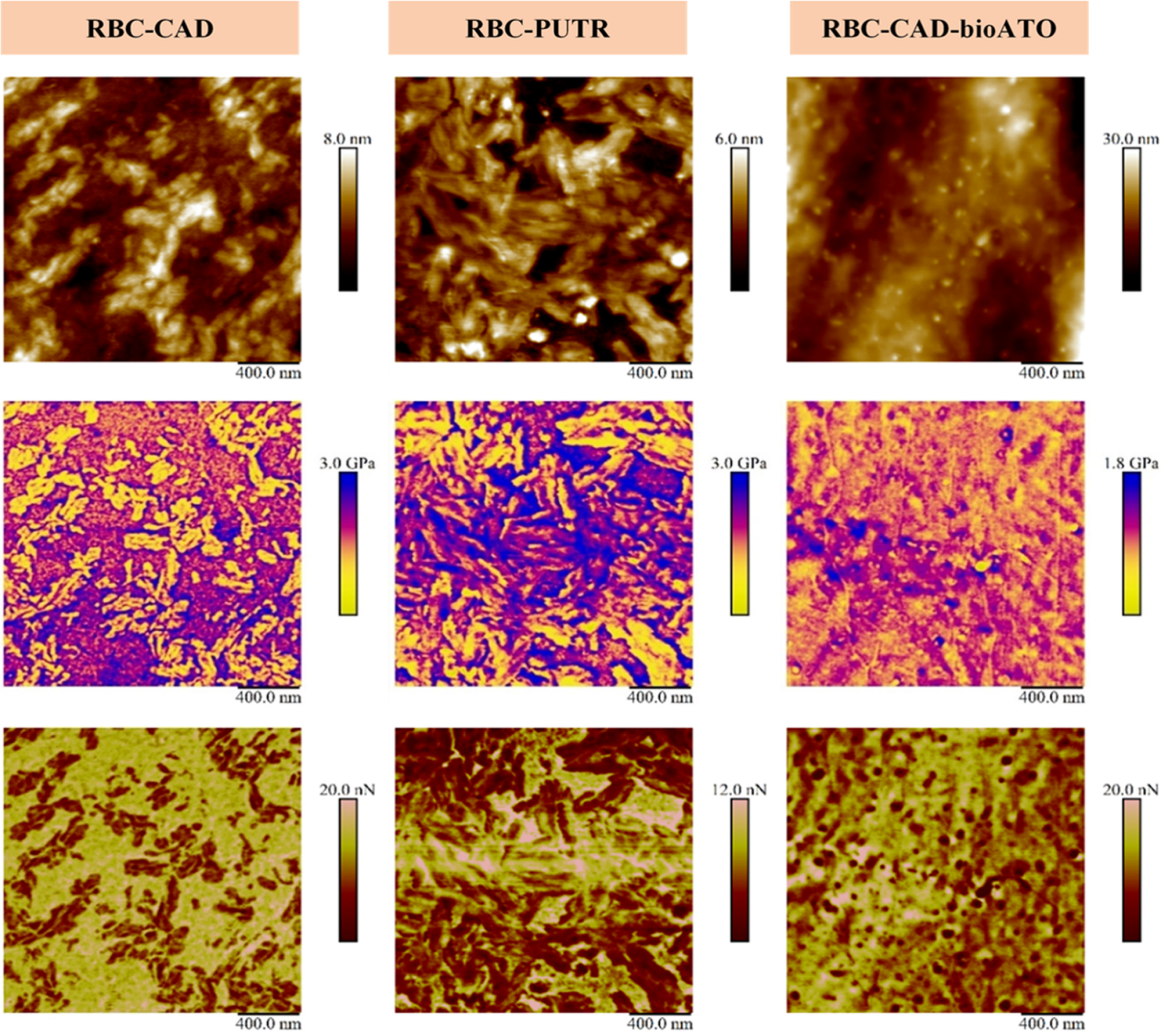
Peakforce QNM height images (top row), DMT modulus (middle row), and adhesion (bottom row) surface maps of RBC–CAD (first column), RBC–PUTR (second column), and RBC–CAD-bioATO (third column).

Moreover, the impact of nanophase separation on the local nanomechanical properties was also investigated by using an AFM-based method, peakforce QNM. This well-known technique can reveal useful information such as elastic modulus, adhesion force and deformation values at the nano-scale level.^59^Figure 3 shows the peakforce QNM topography, DMT modulus, and adhesion images for each NIPU sample.

Even if they were totally amorphous, RBC–CAD and RBC–PUTR exhibited phase-separated morphologies composed of both hard and soft domains. In the contact modulus images of these two samples (second row images of Figure 3), the nanostructures appear as bright colors. This indicates that they have lower elastic modulus (soft domains) whereas the matrix in both cases have dark colors, in line with higher modulus values (hard domains). Besides, these hard and soft domains corresponded to high- and low-adhesive regions, respectively (Figure 3). This emphasizes that the nanostructures in the case of our amorphous NIPUs are likely formed due to the hydrogen bonding between isolated soft domains. They present higher mobility leading to a more ordered structure, whereas the matrix consisted of more rigid disordered domains. Furthermore, the nanostructures in RBC–CAD show needle-like shapes, which were coherent with what was observed in the drop-cast sample but smaller in size and less connected. Like for RBC–PUTR, the soft domains are more concentrated and greater in size. On the other hand, the RBC–CAD-bioATO displays a better homogeneity in terms of mechanical distribution, resulting from the nanophase mixing behavior. This homogeneous distribution can prove that the addition of bioATO as a chain extender can improve the compatibility of separated nanophases compared to RBC–CAD.

**Figure 4:**
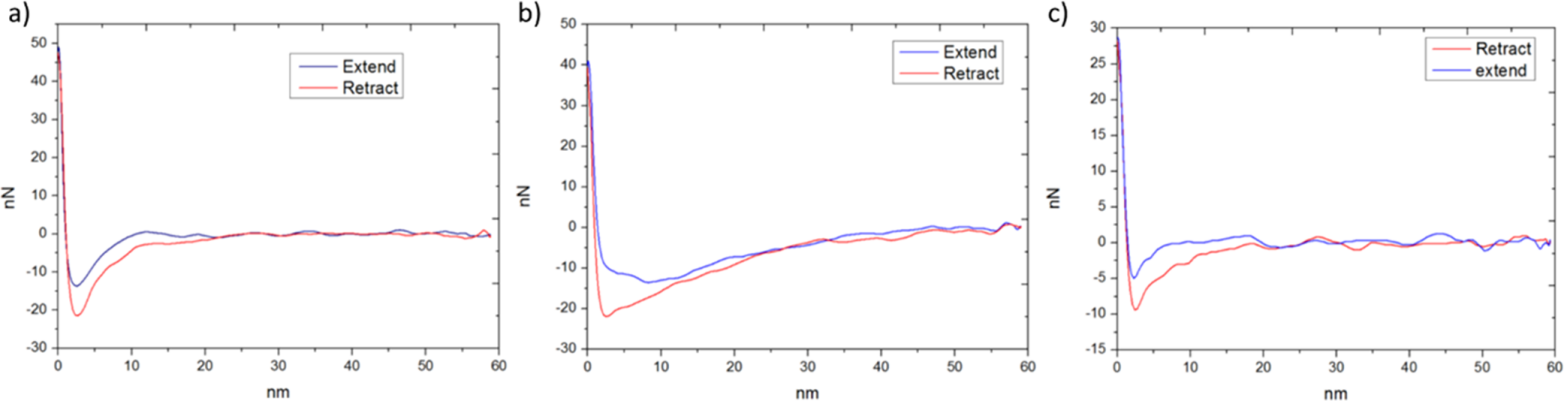
Representative force curves of the NIPU sample, (a) RBC–CAD, (b) RBC–PUTR, and (c) RBC–CAD-bioATO.

Peakforce QNM not only provide qualitative information but can also evaluate the local mechanical properties quantitatively. In the spectroscopy mode, force–deformation curves (FCs) are acquired at each pixel of the images. Figure 4 shows three representative FCs corresponding to each sample.

When the tip is far from the surface samples, the FCs display horizontal, flat lines, which means that there is no interaction between the tip and the sample. As soon as the tip moves close enough to the samples, attractive Van der Waals force pulls the tip toward the surface, leading to negative forces in the FCs. The AFM tip then contacts and indents the surface, causing the force to rise linearly to a positive value. This part of the FCs is called the extend part (indicated by blue color in the figure). After reaching to pre-set force values (peakforce values), the tip starts to retract from the sample and the force slowly decreases again, reaching the lowest negative value (adhesion force). As the interaction between the tip and the sample is lost completely, the force returns to zero. This part of the force curve is called the retract part (indicated by red color). It should be noted that there was no hysteresis loop occurring in the contact part for all samples. This indicates that the tip–sample interactions in all cases are purely elastic, and no plastic deformation has occurred. Therefore, suitable contact mechanic models can be applied to calculate the contact modulus of the samples. The choice of model depends on certain parameters such as the tip shape used to conduct the experiment, the effect of adhesion force, and the deformation types of the materials (plastic or elastic). In our case, the AFM tip used was RTESPA 300-30. This type of AFM probe has a spherical shape with a controlled tip radius of approximately 30 nm and was verified by SEM measurement as shown in Figure S15. Furthermore, the contribution of adhesion forces in each sample was significantly large (20–30 nN). Therefore, the Derjaguin–Muller–Toporove (DMT) model was chosen for the final calculation.^60^Figure S16 shows the boxplot analysis of DMT modulus of RBC–CAD, RBC–PUTR, and RBC-bioATO. The mean values and standard deviation values (*N* = 30) of nanostructures and the matrices were reported. As shown in the corresponding figure, the calculated DMT modulus of the nanostructures and matrix of RBC–CAD are slightly higher than those corresponding to RBC–PUTR. This indicates that changing from CAD to PUTR altered the topological features and had an impact on the local mechanical properties of the NIPU. On the other hand, a homogeneous modulus distribution was obtained in the case of RBC-bioATO, as expected with its nanophase mixing behavior. In addition, the high level of interdomain hydrogen bonding in RBC–CAD-bioATO lowers its local nano-mechanical properties, as previously reported for NIPUs of microphase-separated structure. They show better mechanical properties than phase-mixed ones.^45,61^ This result was confirmed by the DMA (Figure S17, Supporting Information), which demonstrates that RBC–CAD showed higher storage modulus than RBC–CAD-bioATO. It means that the introduction of the chain extender in NIPU architecture increases the flexibility of the system, promoting the chain mobility at room temperature.

### Ferroelectric-like Behavior and Its Origin

**Figure 5:**
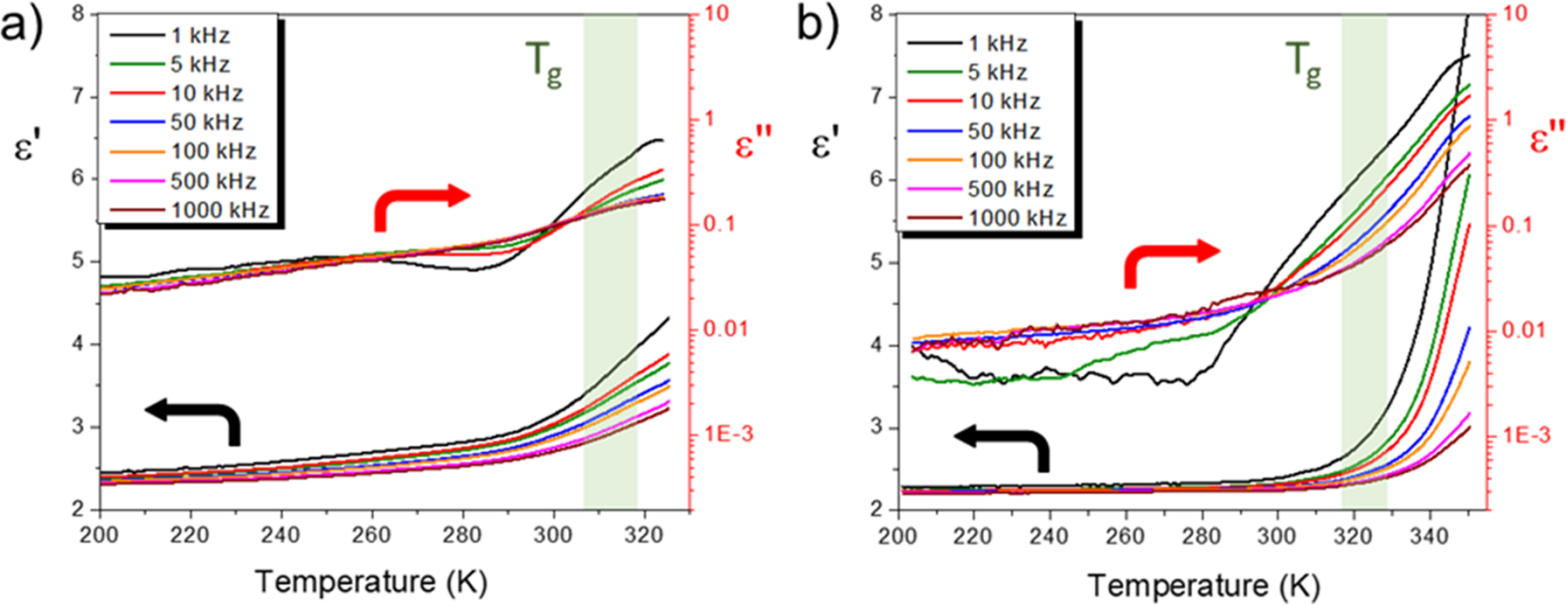
Temperature dependence of the real (ε′) and imaginary (ε″) components of dielectric permittivity during heating at different frequencies for the samples (a) RBC–CAD and (b) RBC–CAD-bioATO. The *T*_g_ region is indicated in both bases.

Dielectric properties of the NIPUs synthesized from RBC with CAD or PUTR were characterized by measuring their complex dielectric permittivity as a function of temperature. Figure 5 shows the temperature dependence of real (ε′) and imaginary (ε″) components of permittivity at different frequencies for RBC–CAD and RBC–CAD-bioATO samples. A similar dielectric behavior was found for samples RBC–CAD (Figure 5a) and RBC–PUTR (included in Supporting Information, Figure S18).

Correspondingly, the imaginary permittivity, related with dielectric losses, shows a distinctive peak/step, more visible in the low-frequency data (<10 kHz) at a temperature that shifts with increasing frequency. This is the typical behavior of the weak dielectric relaxation associated with the glass transition. Note that, above *T*_g_, both permittivity and losses increase sharply, along with higher-frequency dispersion (see also Supporting Information, Figure S19), resulting from the activation of ionic conduction mechanisms. Indeed, low-frequency ac conductivity increases on 1 order of magnitude by only 20 °C, from room temperature to *T*_g_ (Figure S19).

Nevertheless, room-temperature conductivity was rather low in all samples and dielectric losses tan δ were below 0.1, so that high electric fields can be endured. Similar results have been reported for some linear aromatic PUs and polyureas.^24,62^ This dielectric relaxation that takes place at the glass transition is likely associated with the freezing of the hydrogen-bonded dipoles in the amorphous phase below *T*_g_, in agreement with previous reports.^23,24,63^ Note that the low permittivity of these NIPUs (ε′ about 2.4) can be considered advantageous for certain applications such as energy harvesting because high voltage coefficients (g_33_) can result even from low (*d*_33_) piezoelectric coefficients.^2,7^

Despite the apparent similar dielectric behavior of sample RBC–CAD-bioATO (Figure 5b), a couple of differences are worth to be highlighted. First, the permittivity shows negligible temperature dependence and frequency dispersion from −70 °C to room temperature, while dielectric losses were much lower than those of RBC–CAD. Besides, the abrupt increase in permittivity and losses above *T*_g_ were more pronounced for sample RBC–CAD-bioATO. These differences could be associated to the incorporation of the semi-crystalline chain extender, which not only results in lower ionic conduction within the glassy state but also increases mobility of charge carriers above *T*_g_ (Supporting Information, Figure S19). Note also that the dielectric relaxation takes place below room temperature and could be affected by the presence of a second *T*_g_ in this material related to the bioATO chain extender, as shown in DSC analysis (Table 1).

**Figure 6:**
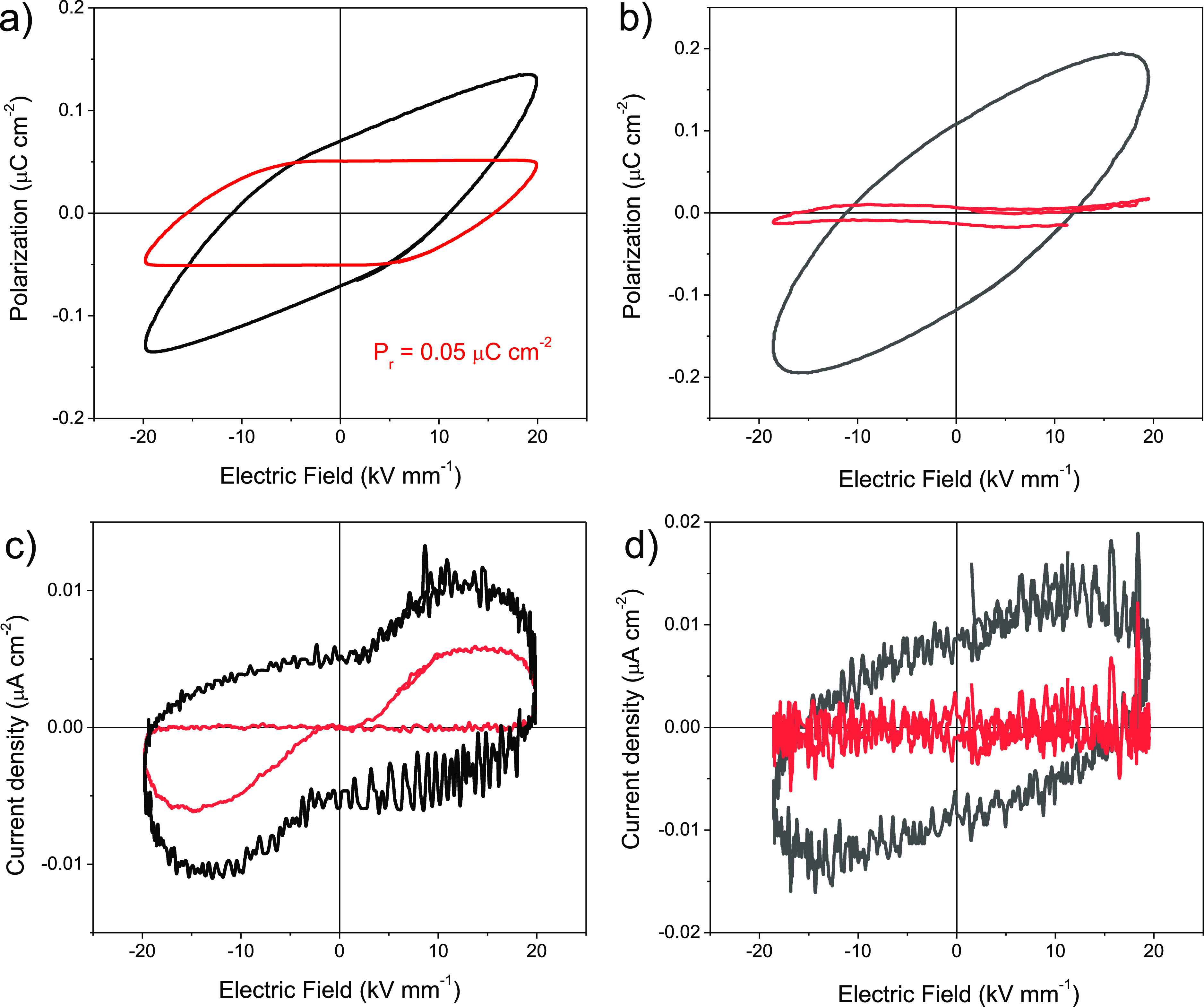
Comparison of P–E hysteresis loops and current density curves as a function of the electric field (I–E) measured at room temperature and 0.01 Hz for RBC–CAD (a,c) and RBC–PUTR (b,d). Red loops are obtained after compensation by subtracting linear polarization and conduction contributions.

Ferroelectric hysteresis loops were measured to set parameters like polarization switching and coercivity; the latter stands for the electric field required to reverse the direction of polar domains/dipoles. Figure 6a,b shows the polarization versus electric field (P–E) hysteresis loops at room temperature for the RBC–CAD and RBC–PUTR samples, for a maximum electric field of 20 kV mm^–1^. Red loops are obtained after compensation by subtracting the linear polarization and conduction contributions, as described in the Experimental section.

A nonlinear behavior of the polarization is clearly shown for sample RBC–CAD with an apparent remanent polarization (*P*_r_) of 0.05 μC cm^–2^. For the RBC–PUTR sample, however, only a linear dielectric-like behavior was found up to 20 kV mm^–1^, and higher electric fields resulted in the sample electrical breakdown.

The ferroelectric-like behavior could be better visualized by analyzing the corresponding current density curves (I–E loops), in which the evolution of the polarization switching can be followed (Figure 6c,d). Note that switching current remains after compensation of I–E loops for sample RBC–CAD (red loop), while no switching current resulted for sample RBC–PUTR. A macroscopic switching seems to take place only in the RBC–CAD sample. It should be mentioned that NIPUs synthesized from RBC with either CAD or PUTR were both amorphous and presented a *T*_g_ slightly above room temperature, so that hysteresis measurements were carried out within the glassy state. This is not the typical case of most known ferroelectric polymers, like PVDF-based ones, which are rather semi-crystalline and typically measured above the glass transition temperature, where polarization switching is favored.^17^ A strategy was also used to obtain ferroelectric switching in some amorphous aromatic PUs, for which P–E hysteresis loops similar to those reported here were found.^20,23^ Therefore, it is important to discuss about the shape and the possible origin of the switching observed in the current loops of these samples to discard the artificial polarizations coming from charge accumulation at interfaces.

The current loops for the RBC–CAD sample are composed of a series of current sparks, which can be even more abrupt as they appear, producing a smoothening of the current due to the large constant time employed in the measurement system. These sparks might come from non-homogeneous switching of polar entities, which can reflect the phase heterogeneity observed in the AFM study of samples (parallel dipole arrangements related to the lamellar-like nanostructuration giving rise to non-cooperative switching). The phenomenon can be observed in amorphous or glassy systems that contain molecular polar dipoles with enough rotational mobility above *T*_g_ but whose mobility is greatly reduced in the frozen glassy state. These systems might suffer from slow switching times due to their high rotational viscosity below *T*_g_.^64^

This behavior is, therefore, different from that of the typical ferroelectrics, which require cooperative switching of the ferroelectric domains/dipoles. Indeed, the loops achieved in sample RBC–CAD resemble rather those typically reported in electrets, where switching comes from mobile charged defects.^64,65^ In ferro-electrets (voided charged polymers), instead, internal charging processes within the cavities of non-polar cellular polymers take place, which can be “switched” or re-charged in the opposite direction via dielectric barrier microdischarges.^66^ Although, NIPUs synthesized from RBC with either CAD or PUTR do not present the typical void structure of cellular polymers but rather a nanophase separation due to hydrogen bonding.

On the other hand, no switching was found in the case of the RBC–PUTR sample, that is, using an even carbon number diamine as a monomer during the NIPU synthesis. This could be explained considering that polar dipoles are disposed symmetrically, resulting in a permanent dipolar moment to be equal to zero. Note also that *T*_g_ of the RBC–PUTR sample is higher than that of the RBC–CAD samples, so that this higher *T*_g_ may result in a worst switching, if any, at room temperature. P–E hysteresis measurements were also carried out at different temperatures for the RBC–CAD sample, as shown in Figure S20 (only loops compensated are given). A similar polar switching behavior was found with decreasing temperature, even at 10 °C, far below the *T*_g_ of the RBC–CAD sample (39 °C), while both *P*_r_ and *E*_c_ apparent values increase with temperature. This seems to indicate better switching performance of the RBC–CAD sample and the role of material’s hardening below *T*_g_, in which the mobility of the polar entities is strongly reduced.

**Figure 7:**
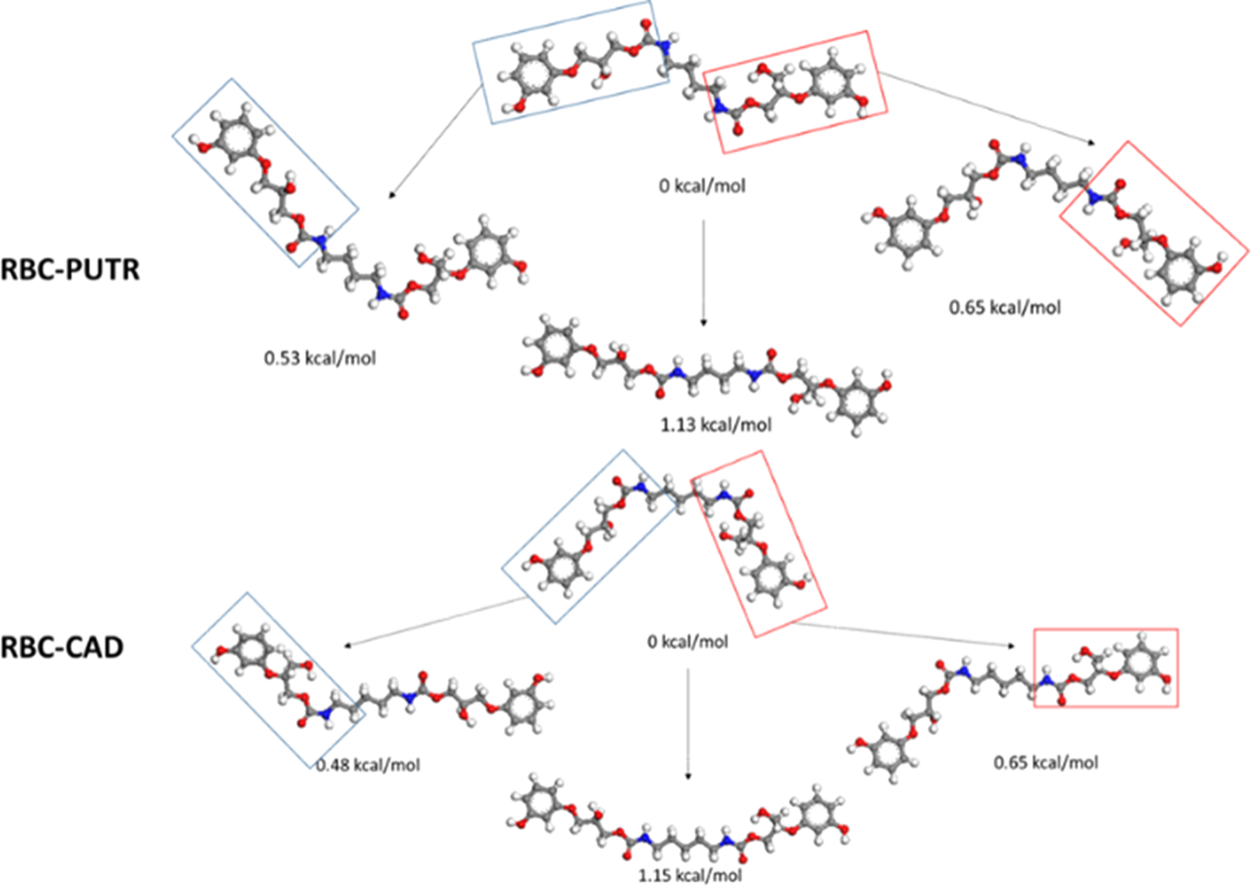
Energetic comparison of the more stable linearized segments in both RBC–PUTR and RBC–CAD.

In order to shed further light on these results, a computational study based on a molecular modeling method was performed to investigate the different chain conformations that can be adopted by our polymers and the interchain hydrogen bonding interactions as a function of the diamine used for the NIPU synthesis. We selected the DREIDING force field61, which describes explicitly the hydrogen bond interactions, for the simulations, as implemented in Materials Studio 18.0.62. First, we have considered the shortest planarized segments of both derivatives. We systematically changed the value of the dihedral angles around the urethane moieties and optimized in each case the backbone with these fixed values. The most stable structures and the linearized chains were finally retained in Figure 7. We adopted a similar strategy for the longer chains in Figure S21.

It is not straightforward to fully address the difference in the level of interactions between chains because the systems are very flexible and are characterized by the presence of stereogenic carbons. Interestingly, when we impose a planarized structure likely imposed by solid-state packing effects, we can distinguish a structural difference between RBC–PUTR and RBC–CAD specimens. In the most stable structure, the chain containing the putrescine as diamine displays the carbonyl groups oriented in opposite directions, while they point in the same direction in RBC–CAD as illustrated in Figure S21. This difference in orientation can potentially influence the pattern of hydrogen bonding between chains, although this pattern can also be influenced by other possible side products obtained during the polymerization. However, these chain conformations are not totally linear but are rather bent, thus representing a non-ideal configuration for the nanostructuration. Accordingly, the more stable linear segments in both NIPUs were also studied (Figure 7). This result confirms that one of the factors that could explain the polar switching response of RBC–CAD is the presence of a net dipole moment due to the parallel dipoles formed by the carbonyl groups throughout the polymeric NIPU chain. Similar results were already reported in the case of polyureas, polyamides, and more recently some PUs. Indeed, it was reported that dipoles in odd nylons such as nylon 11 and nylon 9 are oriented in the same direction, whereas in even nylons, the net dipole moment becomes zero because dipoles are arranged in an antiparallel fashion.^67,68^ Similarly, it was reported that even linear aromatic PUs have antiparallel dipoles, resulting in a non-polar state, while odd linear aromatic PUs have parallel dipoles and are thus polar.^24^ Therefore, it is possible to conclude that the RBC–CAD obtained from an odd carbon number diamine is in a polar state with a net dipole moment.

**Figure 8:**
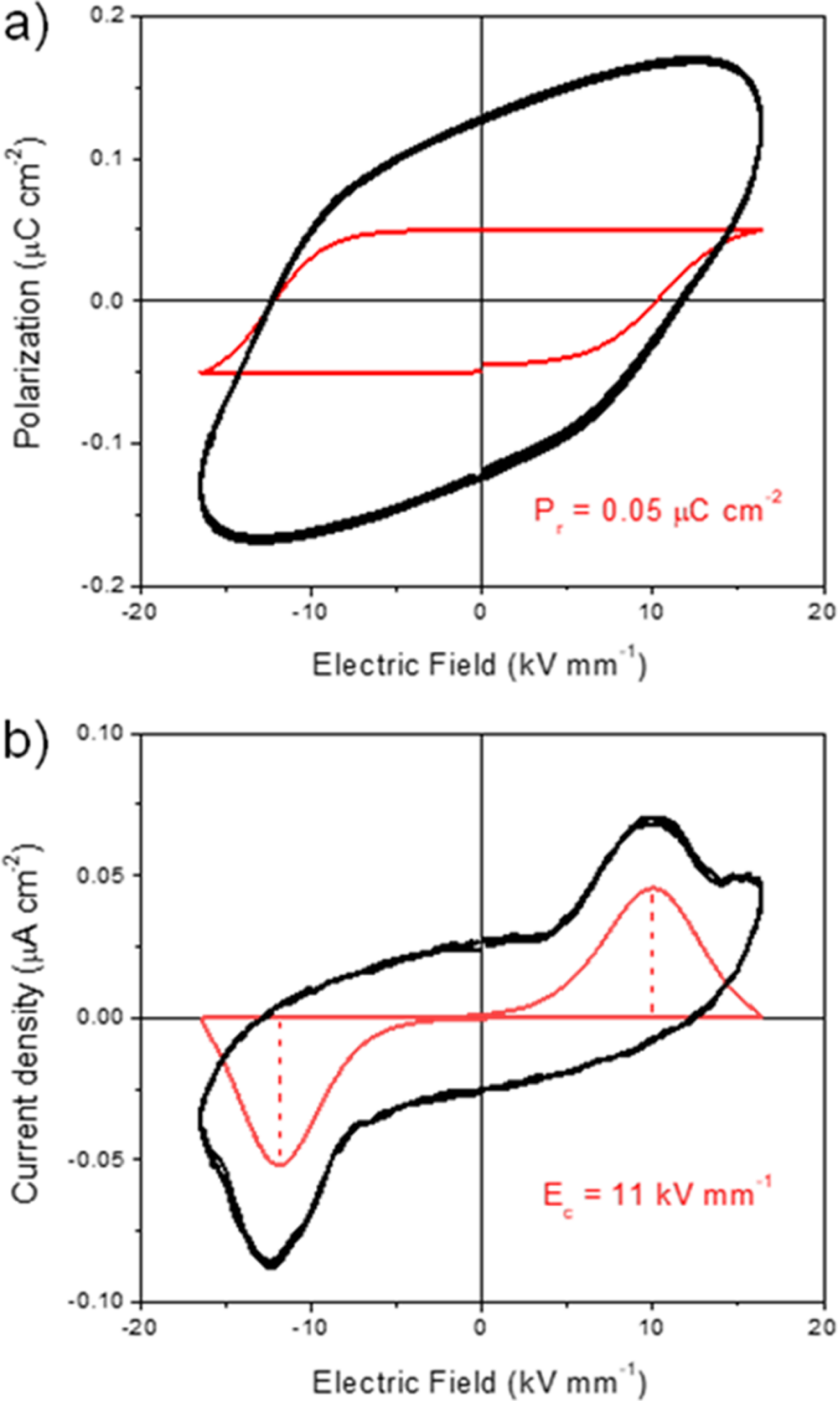
Room-temperature P–E hysteresis loop (a) and current density curve for RBC–CAD-bioATO (b) measured at 0.01 Hz. Red loops are obtained after compensation by subtracting linear polarization and conduction contributions.

Nevertheless, having dipole moments does not necessarily mean having the ferroelectric-like behavior, for which cooperative switching of polar entities/dipoles is required. We then study the ferroelectric switching behavior of RBC–CAD-bioATO to determine the effect of the greater flexibility and mobility of the chains due to the presence of the semicrystalline chain extender. Figure 8 shows the room-temperature P–E hysteresis and current density loops for RBC–CAD-bioATO. In this case, red loops are obtained after compensation by subtracting not only the linear polarization and conduction contributions but non-linear leakage currents were also considered.

It should be noted that, contrary to the RBC–CAD sample, both polarization and current density curves for the RBC–CAD-bioATO sample clearly indicate cooperative switching, reminiscent of the typical response of ferroelectric material. A *P*_r_ of 0.05 μC cm^–2^ resulted in this case in an *E*_c_ of 11 kV mm^–1^, figures lower than those found for, for example, PVDF-based polymers.^13,17^ The lower coercive fields are advantageous for practical applications.

Note that bioATO introduces some semi-crystallinity into the NIPU and that a second *T*_g_ was found at room temperature associated with it in sample RBC–CAD-bioATO (Figure S9), so that ferroelectric switching would take place in a softened environment. Ferroelectric switching performance observed when bioATO is added to RBC–CAD-based NIPU could be related to the enhanced compatibility of the soft and hard phases observed by AFM, which indicated a more homogeneous morphology and a better mechanical coupling between them. These results evidence the ferroelectric-like behavior of this fully biobased NIPUs produced by the solvent-free process.

The Berlincourt piezoelectric coefficient *d*_33_ was evaluated after hysteresis measurement, in which poling of the samples was attained by turning off the electric field right before completing the loop at a low measuring frequency. NIPUs synthesized from RBC with CAD do not show *d*_33_ activity in Berlincourt, but a meaningful *d*_33_ value of 2 pC N^–1^ resulted for the poled RBC–CAD-bioATO sample, the one that showed loops with clear indications of ferroelectricity. This value must be compared with those reported for (non-PVDF) polymers, such as nylons, polyamides, cellulose, and their derivatives or PUs, for which effective piezoelectric responses between 2 and 5 pC N^–1^ are typically reported.^2,69^ Note that, despite the low *d*_33_ achieved, high voltage coefficients g_33_ (above 0.1 V m N^–1^) are anticipated due to the very low permittivity of NIPUs, although corresponding figures of merit for energy harvesting applications are less than desired.^70^

Nevertheless, these materials can be processed in large areas to produce enough power for applications. A more efficient poling is needful, which requires tailored conditions like poling above *T*_g_ and/or higher electric fields, where electrical breakdowns are a drawback. Conductivity values would need to be thus optimized. To improve the rotational mobility within the highly viscous glassy state is necessary for a better stability of the aligned polar entities, a critical challenge for functionality in energy harvesting applications.

## Conclusions

Fully biobased NIPUs have been successfully synthesized using a solvent-free process. REX allowed to synthetize NIPUs by avoiding the difficulties related with heat and mass transport due to the high viscosity, typically observed in their batch-type syntheses. The molecular architecture of the obtained NIPUs has been designed to show a final polar structure and consequently to get the ferroelectric-like behavior. Using resorcinol bis-carbonate and cadaverine as monomers, a NIPU characterized by high *T*_g_ and a permanent dipolar moment has been obtained. Although the obtained NIPUs were totally amorphous, AFM characterization showed their phase-separated nanostructurations. Depending on the processing method, RBC–CAD showed different nanostructuration, as ordered needle and lamellar nanostructure, when it was processed by drop casting method and by compression molding, respectively. The nanophase separation has been ascribed to the high level of interurethane hydroxy hydrogen bonding present in the hard segment of NIPUs as it was evidenced by performing AFM as a function of temperature. Adding a biobased diamine oligomer as a chain extender, the flexibility of the molecular architecture was increased and the phase compatibility as well, maintaining the polar structure. Macroscopic ferroelectric switching was achieved for sample RBC–CAD-bioATO, and piezoelectric activity demonstrated to be a behavior that can be tuned by changing the NIPU molecular architectures, being potential candidates for emerging applications like in energy harvesting and can enable the design of self-powered devices such as follow-up medical devices, despite the low piezoresponse obtained (2 pC N^–1^). We believe that these materials afford a real response to the large demand to implement sustainable energy harvesters, while affording environmentally friendly materials. Moreover, RBC–CAD and RBC–CAD-bioATO are promising materials for the processing of biobased polymer–ceramic composites with enhanced piezoelectric response, being a greener and sustainable alternative to piezoceramics.

## Supporting Information Available

Experimental section, REX, FTIR spectra, ^1^H NMR spectra, thermal properties and stability, morphology by AFM, local mechanical properties by AFM and DMA, ferroelectric properties, and computational study (PDF)
