# Supplementary material for: Solvent-Free Design of Biobased Non-isocyanate Polyurethanes with Ferroelectric Properties
Source: ACS Sustain Chem Eng. 2021 Oct 27;9(44):14946–58. doi: 10.1021/acssuschemeng.1c05380 (PMC8579420; doi:10.1021/acssuschemeng.1c05380)
Supplement: Supplementary file 1 — sc1c05380_si_001.pdf [file sc1c05380_si_001.pdf]

Supplementary information

Solvent-free design of biobased non-isocyanate polyurethanes with ferroelectric properties.

Valentina Sessini,† Cuong Nguyen Thai, ‡,^ Harvey Amorín,§ Ricardo Jiménez, § Cédric Samuel,‖ Sylvain Caillol,∇ Jérôme Cornil,‡ Sébastien Hoyas,‡,○ Sophie Barrau,^ Philippe Dubois,† Philippe Leclère,‡, and Jean-Marie Raquez*†*

† Laboratory of Polymeric and Composite Materials, Center of Innovation and Research in Materials and Polymers (CIRMAP), University of Mons – UMONS, Place du Parc 23, 7000 Mons, Belgium.

‡ Laboratory for Chemistry of Novel Materials (SCMN), Center of Innovation and Research in Materials and Polymers (CIRMAP), University of Mons – UMONS, Place du Parc 23, 7000 Mons, Belgium.

^ Université de Lille, CNRS, INRAE, Centrale Lille, UMR 8207 - UMET - Unité Matériaux et Transformations, F-59000 Lille, France.

§ Instituto de Ciencia de Materiales de Madrid (ICMM), CSIC, Cantoblanco, 28049 Madrid, Spain.

‖ IMT Lille Douai, Institut Mines-Télécom, Univ. Lille, Centre for Materials and Processes, F-59000 Lille, France.

[∇] ICGM, Université de Montpellier, CNRS, ENSCM, UMR 5253 - Place Eugène Bataillon CC 1700 - Bâtiment 17, Montpellier, France

[O] Organic Synthesis & Mass Spectrometry Laboratory, Interdisciplinary Center for Mass Spectrometry (CISMa), Center of Innovation and Research in Materials and Polymers (CIRMAP), University of Mons – UMONS, Place du Parc 23, 7000 Mons, Belgium.

Table of Contents

1. **Experimental section**
2. **REX**
3. **FTIR spectra**
4. **^{1}H-NMR spectra**
5. **Thermal properties and Stability**
6. **Morphology by AFM**
7. **Local mechanical properties by AFM**
8. **DMA**
9. **Ferroelectric properties**
10. **Computational study**

1. Experimental section

Materials

Resorcinol biscyclecarbonate (RBC) (310.26 g/mol) was made as reported elsewhere[1] and used without any further purification. 1,5-diaminopentane (CAD) (102.18 g/mol) and 1,4-diaminobutane (PUTR) (88.15 g/mol) were supplied from Alfa Aesar and used as received. Deuterated Dimethylsulfoxide (DMSO-d_6) was purchased from Sigma Aldrich (Belgium). The synthetized Amino-Telechelic Oligoamide (bioATO) (3784 g/mol) was kindly supplied by Materia Nova Research Center. Its synthesis was performed as reported elsewhere.[2] Briefly, dimer fatty acid Pripol 1013 and 1,2-diaminoethane were mixed together with a [NH_2] to [CO_2H] molar ratio of 1.2 at 160 °C. After 1 h, the reaction temperature was increased until 190 °C for 6 h. At the end of the reaction, the mixture was cooled down at room temperature, so obtaining a rigid yellow material.

Synthesis procedure

The liquid monomers, were weighed at a stoichiometric ratio of 1 and manually premixed in order to activate the aminolysis reaction and obtain a solid material in a few minutes, thus facilitating its injection in the microcompounder and avoiding any losses of material. When bioATO were used as chain extender, the stoichiometric ratio was maintained (RBC:CAD:bioATO – 1:0.66:0.33). The solvent free synthesis was performed using a twin-screw DSM microcompounder (Xplore Instruments BV) for 1 hour at 100 °C. The rotation speed of the twin screw was kept constantly at 100 rpm. The uniaxial force, temperature and pressure were monitored by using the Xplore software. The samples were finally processed by compression molding at 100 ºC under a pressure of 5.5 Pa for 3 minutes. Subsequently, they were cooled down slowly until room temperature for 10 minutes.

Characterization methods

The main reactant and the obtained NIPUs dissolved in deuterated Dimethyl Sulfoxide DMSO-d_6 (10 mg/mL) were analyzed by ^1H NMR on a Bruker AMX-500 (500MHz) at 25°C.

FTIR spectra were recorded on an ATR-mode Bruker Tensor 17 spectrometer. Spectra were recorded in transmission mode in the range of 4000–600 cm^{-1} with a nominal resolution of 4 cm^{-1}.

The thermal characterization was performed by dynamic differential scanning calorimetry (DSC) analysis and thermogravimetric analysis (TGA). DSC measurements were carried out using DSC Q200 TA Instruments with heat/cool/heat program in the range of -80 to 150 °C with a heating/cooling rate of 10 °C/min under nitrogen purge (50 mL/min). The glass transition temperature (T_g) was calculated from the second heating scan and it was taken at the mid-point of heat capacity changes. The melting temperature (T_m) and crystallization temperatures (T_c) were obtained from the second heating and cooling scans, respectively.

The TGA measurements were carried out to study the thermal decomposition behavior using a TA-TGA Q500 analyser. A 10 mg weight sample was heated from room temperature to 550 °C at a heating rate of 10 °C/min under nitrogen atmosphere (60 mL/min).

Dynamic Mechanical Thermal Analysis (DMTA) of the samples was carried out using a DMA Q800 from TA Instrument on film tension mode with an amplitude of 5 μm, a frequency of 1 Hz, a force track of 125 %, and a heating rate of 3 $°C·min^{-1}$. Samples subjected to DMTA were cut from compression-molded thin films into regular specimens of approximately 20 mm × 5 mm × 0.50 mm.

The morphology of our samples was studied by atomic force microscopy (AFM). All of the materials were cut into specimens with a size of 1x1 cm and were then attached onto a steel disk by double-sided tapes. All experiments were carried out by Bruker Dimension Icon (X-Y scan range: 90μm x 90μm; Z range: 10μm) with Nano Scope V as Controller. The software used to process and analyse the images was NanoScope Analysis 1.8. The scan rate was 0.5 Hz and the number of data points in X and Y was kept constantly at 512. The tip used for the Tapping AFM is produced by Bruker Company with a spring constant of 40 N/m and a resonant frequency of 325 kHz.

There are two types of data given in Tapping AFM: Height and Phase. On one hand, Height image will give quantitative information about the morphology of the samples and the scale is presented by a color bar. The brighter the color is, the

higher the structure is and vice versa. One the other hand, phase image will give qualitative information about the composition of the sample.
The thermal stability of the nanostructured morphology was investigated. The sample was placed into a glass substrate by a double-sided tape and then positioned on top of a magnetic support. Finally, the whole system was put on a heating stage. The temperature was precisely controlled by a heat controller with a cooling system with water circulation. The temperature of the heating state and the AFM scanner head was monitored in real-time. During the experiment, the temperature of the AFM scanner head was constantly stable at 25 °C. The procedure for the experiment was as follows: First, Tapping AFM was deployed to characterize the initial morphology of the sample at room temperature (25 °C). Then, the morphology was observed at different elevated temperatures in order to check the evolution of the nanostructures until 75 °C. More precisely it was checked every 5 °C followed by 10 min of stabilization after rising the temperature.

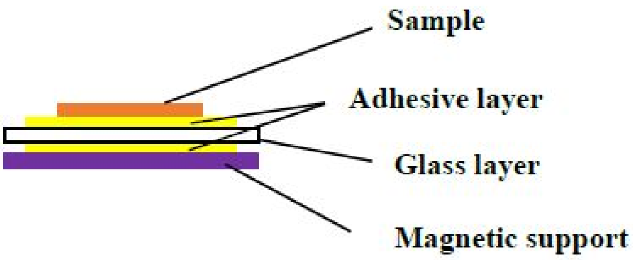

Scheme S1. The preparation of the sample for the experiment.

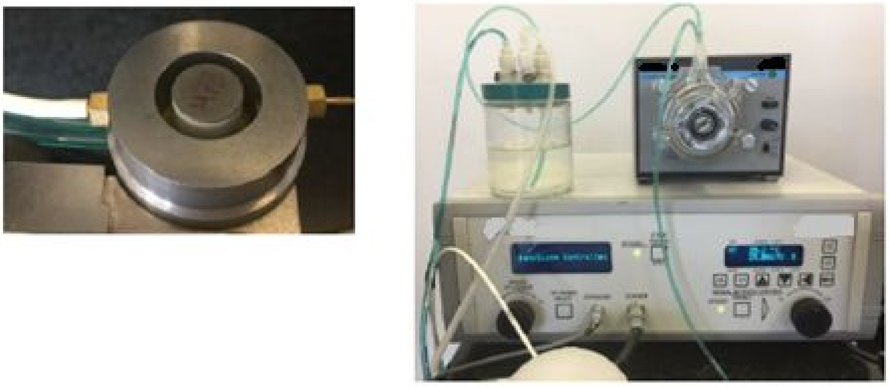

Scheme S2. The heating stage (left) and heating controller system (right).

For the investigation of the local mechanical properties, AFM analysis on PFQNM mode was used. On this mode, both topological features and local mechanical properties of the sample, such as Young’s modulus as well as adhesion, can be

mapped simultaneously. The AFM tips used for the experiment, RTESPA-300-30, were supplied by Bruker Company and have a nominal spring constant of approximately 40 N/m with a tip radius of 30nm. Prior to the experiment, the deflection sensitivity of the cantilever was determined by the linear ramp method using a standard, sapphire disk (Bruker Company). For all samples, the experimental parameters were set as follows: the peak force was varied between 30 - 50 nN to keep a sufficient indentation depth of 2 - 3 nm, the peak force amplitude was 30 nm, the modulation frequency was 2 kHz and the scan rate was 0.5 Hz. The nanomechanical mapping images of each sample (contact modulus, adhesion force and dissipation energy) was analysed by Nanoscope 1.8 Software.

Electrical characterization was carried out on NIPU films (~65 μm thickness) metallized using Ag electrodes (7 mm in diameter) deposited by vacuum sputtering. The temperature dependence of dielectric permittivity and losses was measured during heating/cooling cycles at 1.5 °C min^{-1} rate and several frequencies between 100 Hz and 1 MHz, using a precision LCR Meter (HP4284A Agilent). A Cryostat Janis VPF 700 coupled to a temperature controller Lakeshore 331 and vacuum condition was used.

Ferroelectric P-E and I-E hysteresis loops were obtained by current integration method. Low-frequency (0.01-1 Hz) and high-voltage sine waves were applied by the combination of a synthesizer/function generator (HP3325B, Hewlett Packard) and a high-voltage amplifier (Trek Model 10/40A). Surface charges were measured with an in-house made charge-to-voltage converter. Ferroelectric-type loops are presented after compensation by subtracting the linear polarization and conduction contributions, by assuming a resistance and a capacitance in parallel.[3] For each applied electric field, several high-voltage cycles (3 to 5 cycles) were performed to ensure a stable switching. A high-voltage poling was then accomplished under the maximum electric field attained, by removing the field just before completing the loop. The d_{33} piezoelectric response was evaluated after the hysteresis characterization with a Berlincourt piezometer (Channel Products Inc).

2. REX

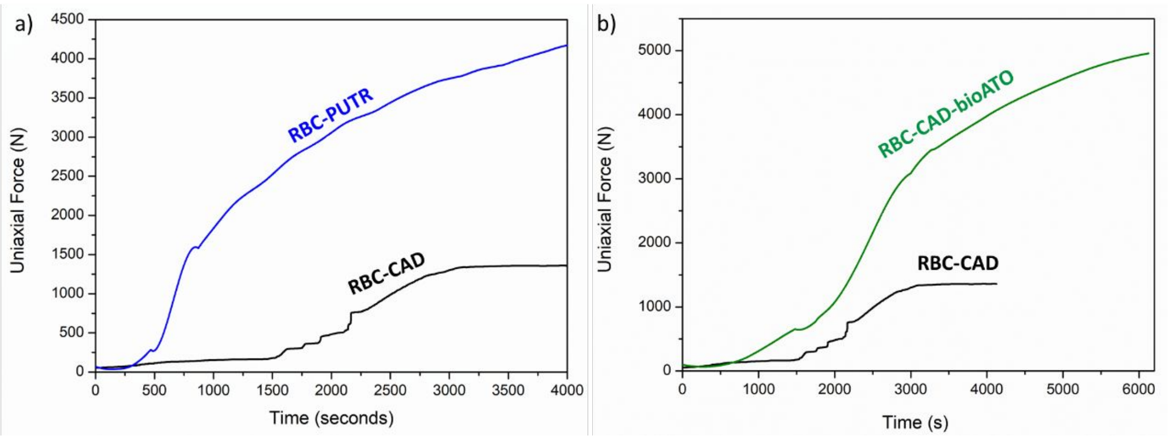

Figure S1. Uniaxial force in function of time during REX.

3. FTIR spectra

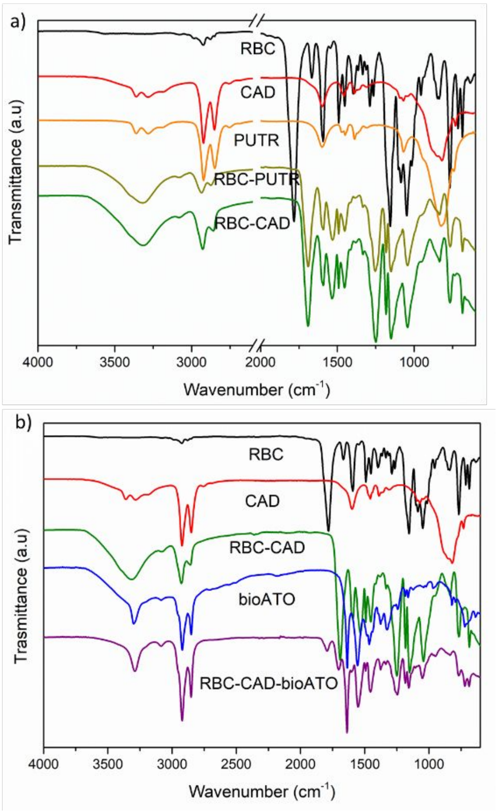

Figure S2. FTIR spectra of the corresponding monomers and the obtained biobased NIPUs. a) Comparison between RBC-CAD and RBC-PUTR systems. b) Comparison between RBC-CAD and RBC-CAD-bioATO systems.

4. ^{1}H-NMR spectra

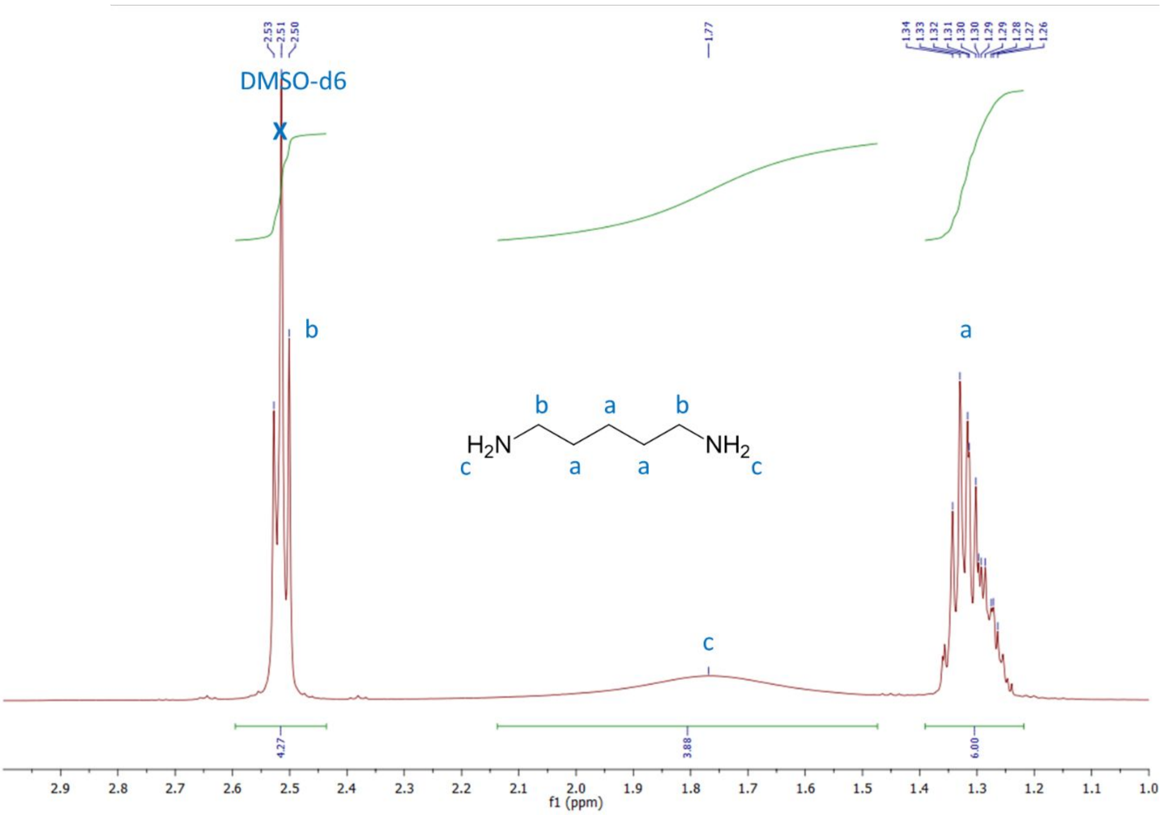

Figure S3. ^{1}H NMR spectrum of the 1,5 diaminopentane (CAD) in DMSO-d_6.

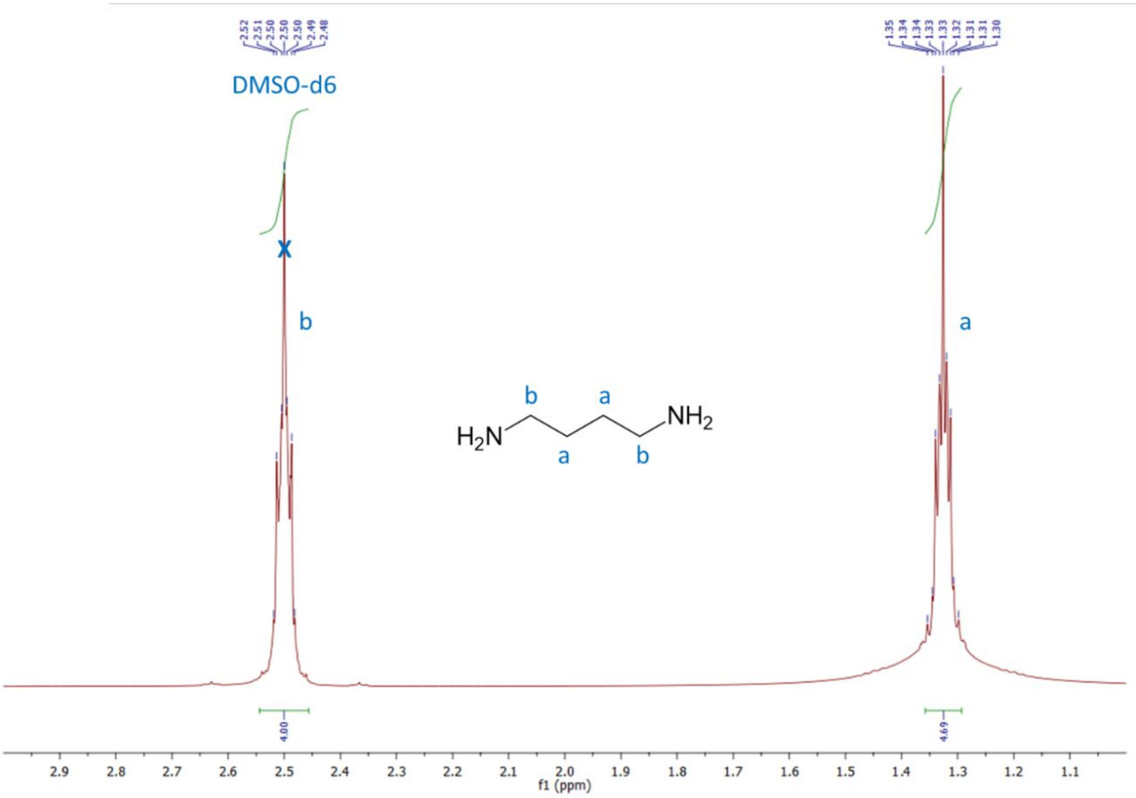

Figure S4. ^{1}H NMR spectrum of the 1,4 diaminobutane (PUTR) in DMSO-d$_6$.

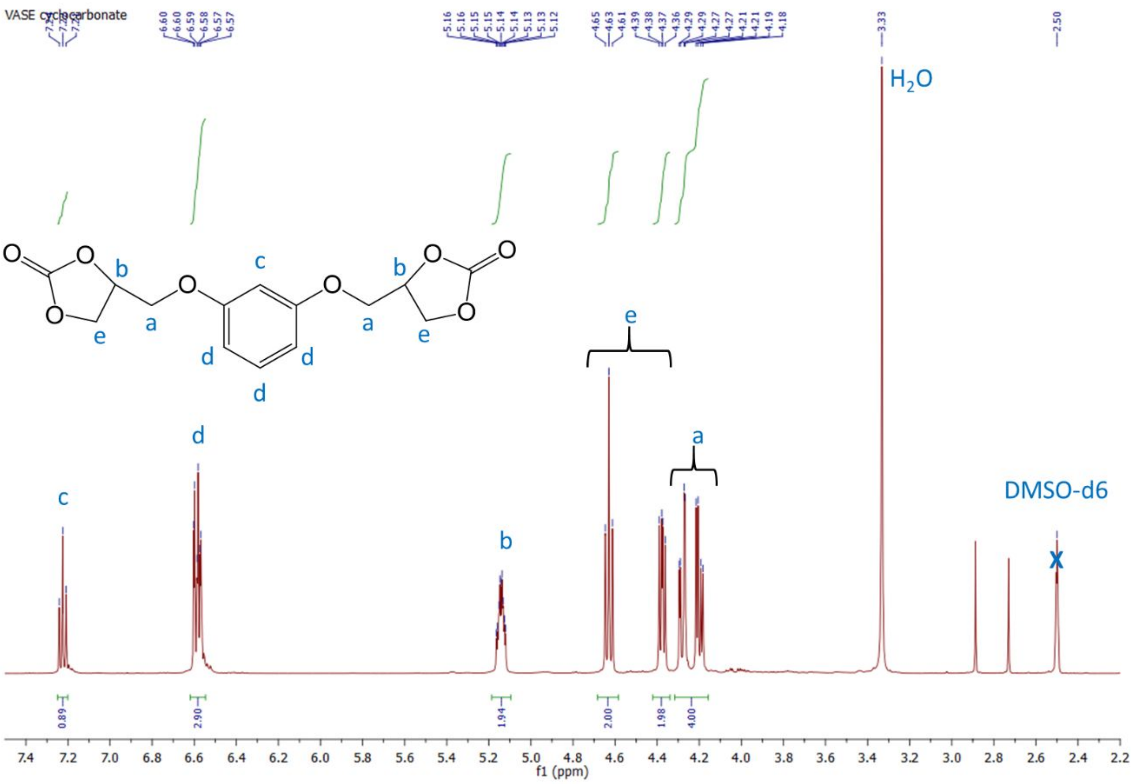

Figure S5. ^{1}H NMR spectrum of the resorcinol bicyclic carbonate in DMSO-d$_6$.

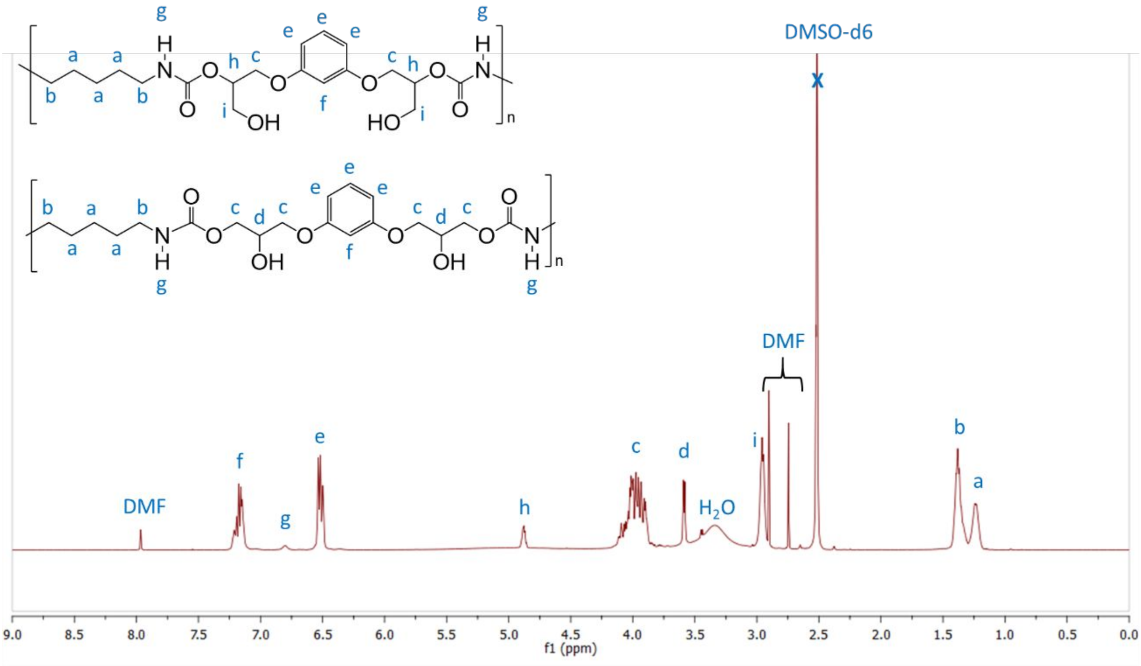

Figure S6. ^{1}H NMR spectrum of the RBC-CAD in DMSO-d$_6$. Product ratio (2º OH: 1º OH) 69:31.

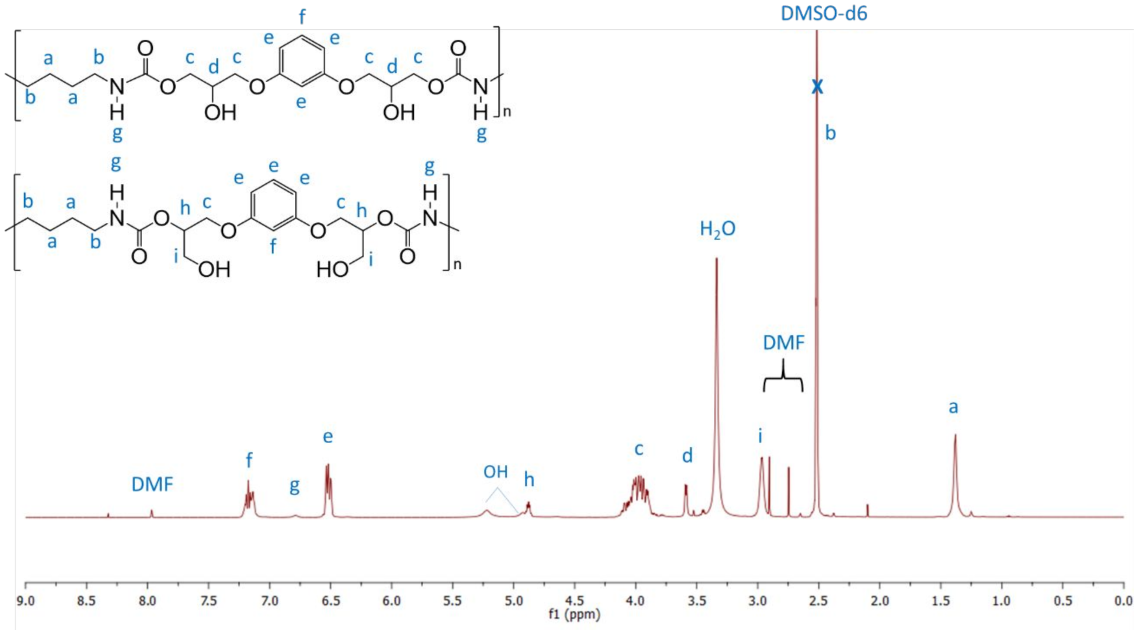

Figure S7. ^{1}H NMR spectrum of the RBC-PUTR in DMSO-d$_6$. Product ratio (2º OH: 1º OH) 64:36.

5. Thermal properties and stability

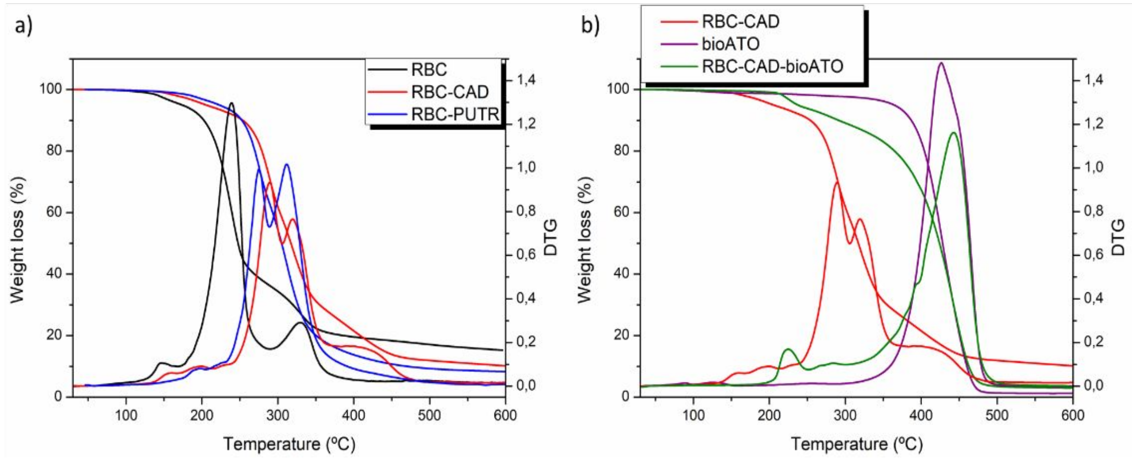

Figure S8. Thermograms and derivative thermograms of the NIPUs, RBC and bioATO.

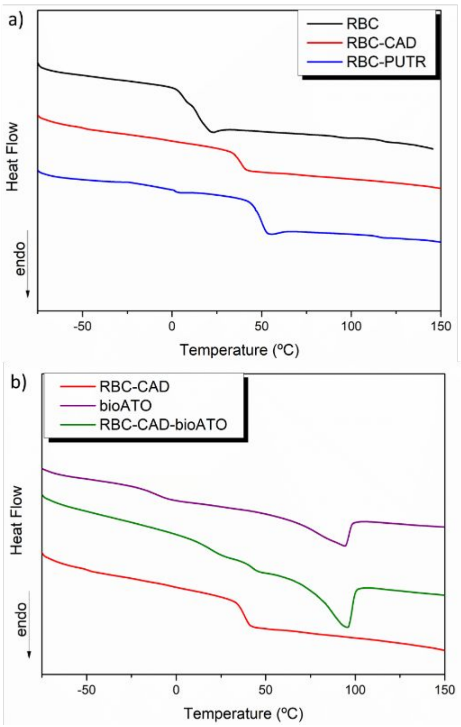

Figure S9. DSC second heating scan for all the synthetized NIPUs. a) comparison between odd and even carbon number NIPU and b) comparison between RBC-CAD and its respective NIPU with bioATO as chain extender.

6. Morphology by AFM

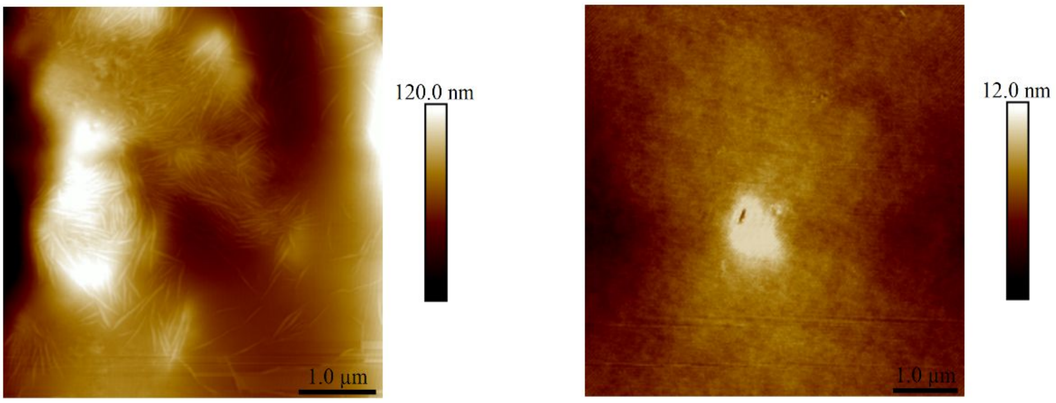

Figure S10. Morphology of RBC-CAD (left) and RBC-PUTR (right) prepared by drop casting.

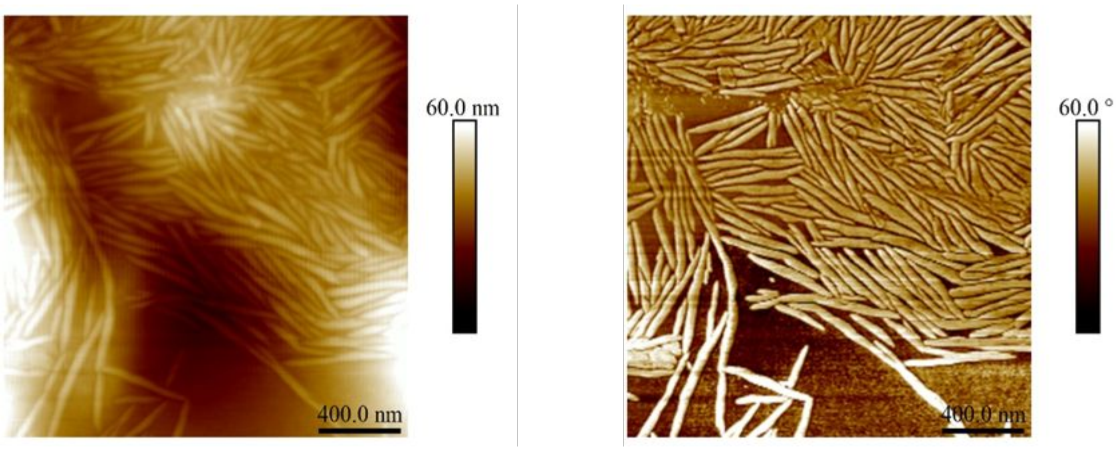

Figure S11. AFM Height (Left) and Phase (Right) images of RBC-CAD prepared by drop casting with higher magnification.

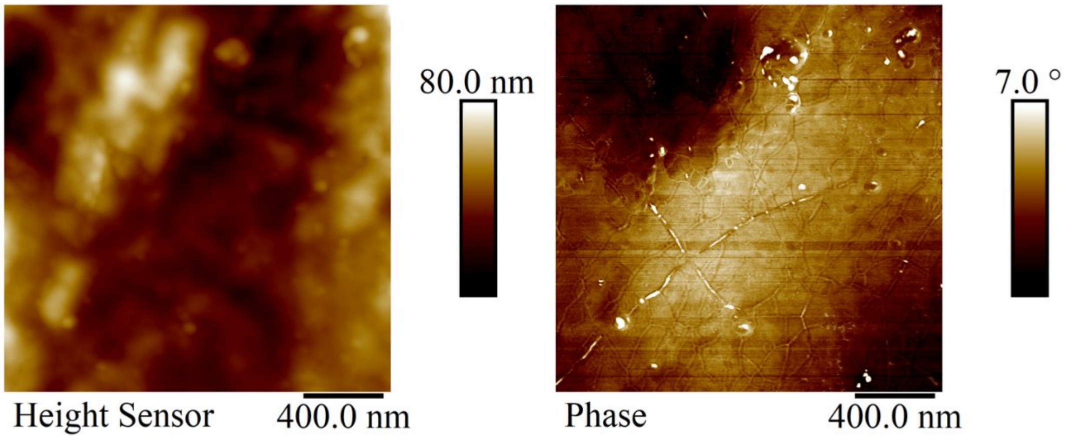

Figure S12. AFM Height (Left) and Phase (Right) images of RBC-CAD-bioATO prepared by compression molding.

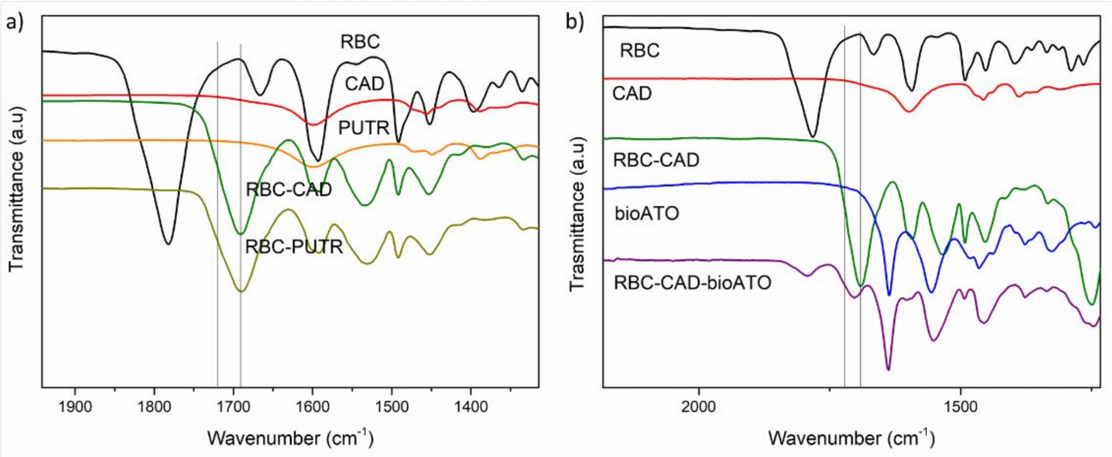

Figure S13. Comparison of FTIR NIPUs spectra in the urethan carbonyl region. Free non-hydrogen bonded carbonyl appears at ~1720 cm^{-1} while hydrogen-bonded carbonyl appears at ~1690 cm^{-1}.

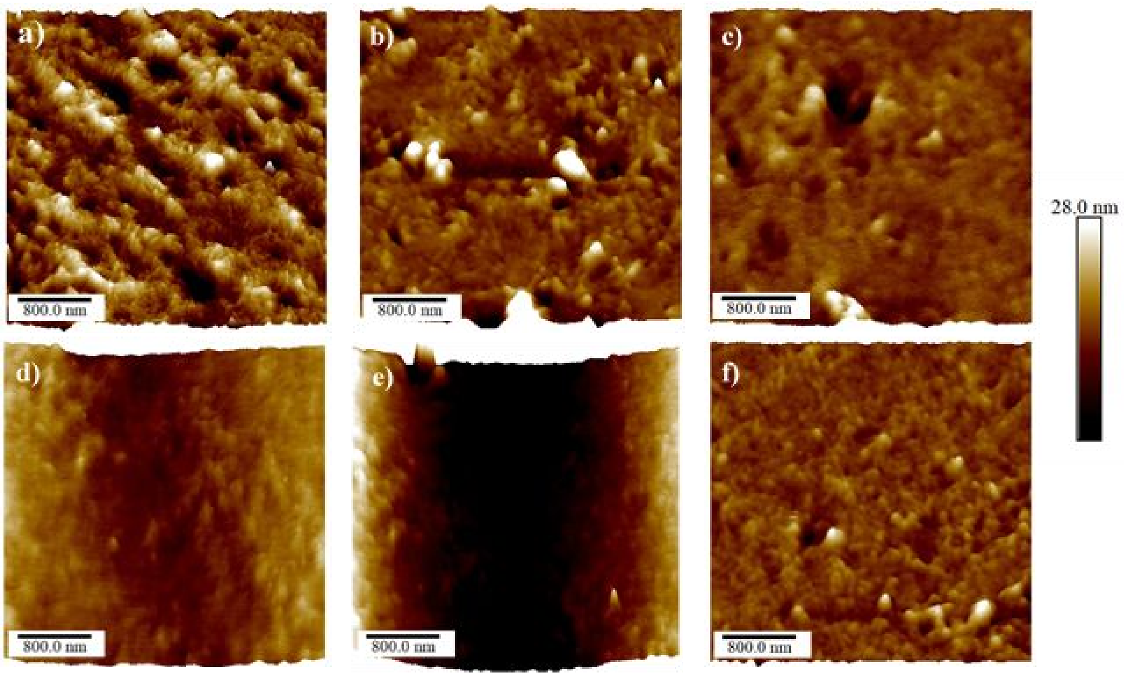

Figure S14. Evolution of RBC-CAD nanostructure with respect to temperature:

a) 25 ºC; b) 40 ºC; c) 55 ºC; d) 70 ºC; e) 75 ºC and f) 25 ºC (Cooling).

7. Local mechanical properties by AFM

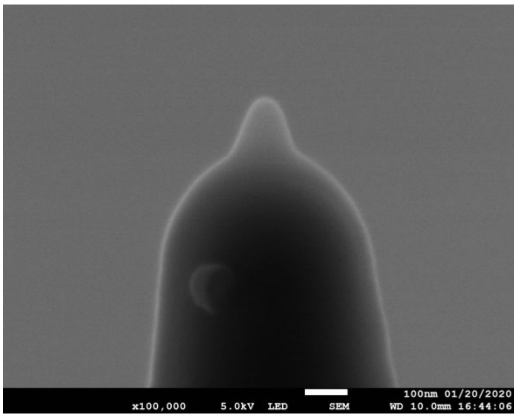

Figure S15. SEM images of RTESPA-300-30 tip. From the image, the tip can be considered as spherical with a radius of approximately 30nm.

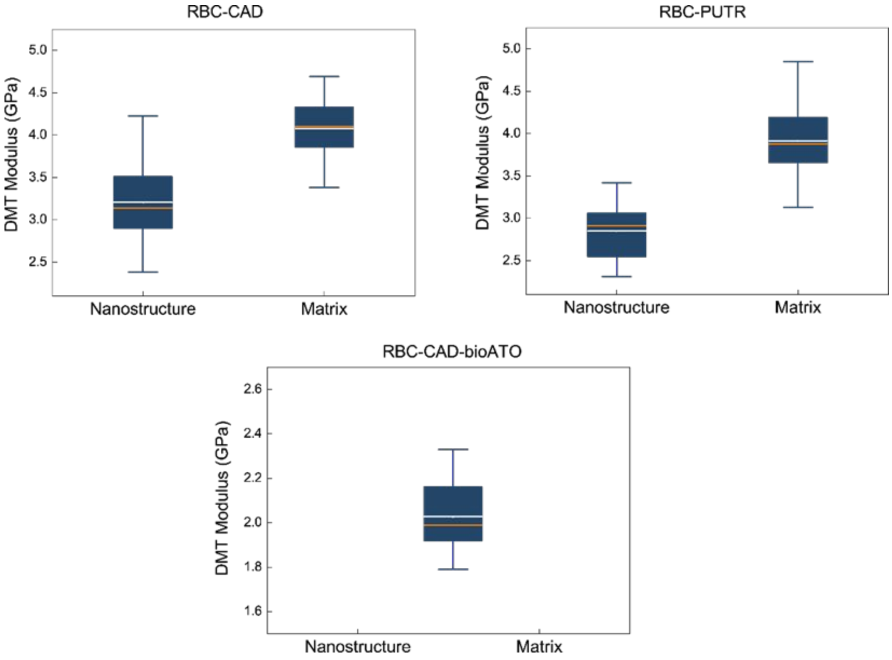

Figure S16. Boxplot analysis of contact modulus of RBC-CAD, RBC-PUTR and RBC-CAD-bioATO. The box-splitting horizontal, black line gives the median values while the orange line gives the mean values, the top and bottom black lines give the maximum and minimum of the measured modulus values.

8. DMA

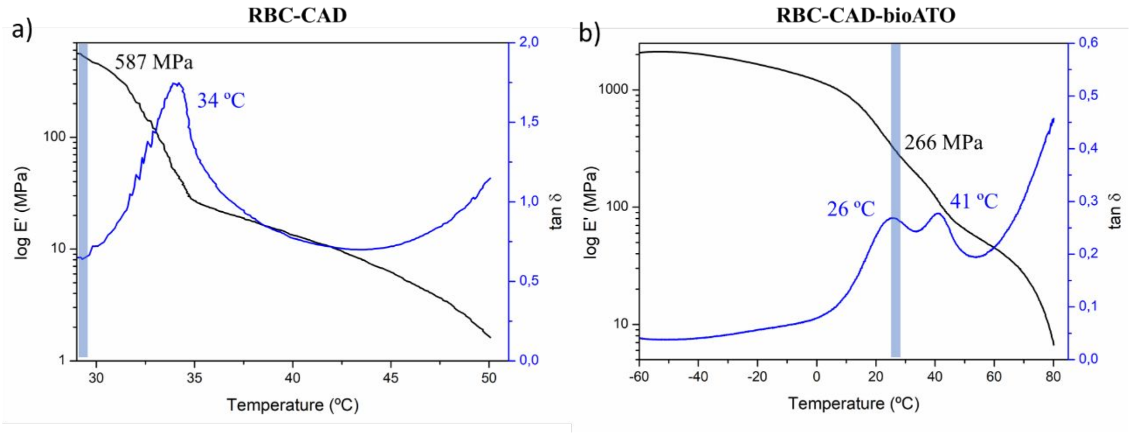

Figure S17. Dynamic mechanical thermal analysis, evolution of the storage modulus and tan δ in function of the temperature for: a) RBC-CAD and b) RBC-CAD-bioATO.

9. Ferroelectric properties.

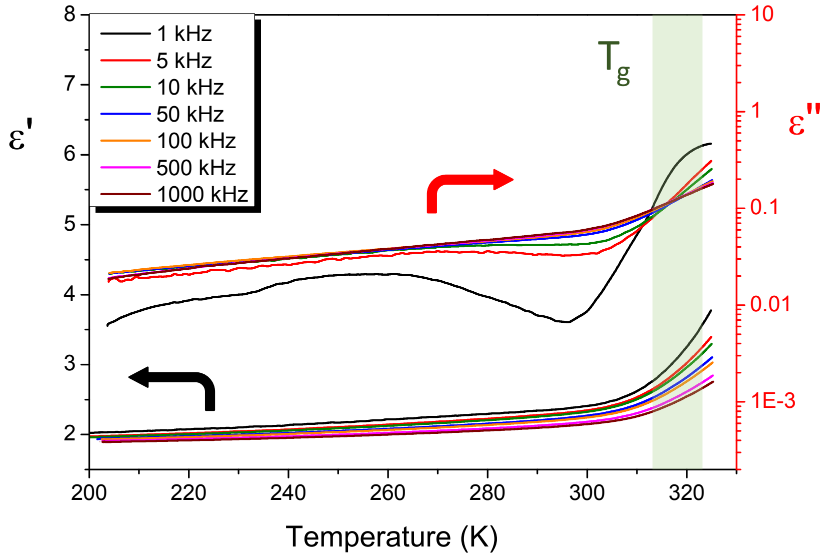

Figure S18. Temperature dependence at different frequencies of the dielectric permittivity and losses of RBC-PUTR sample. T_g region is indicated.

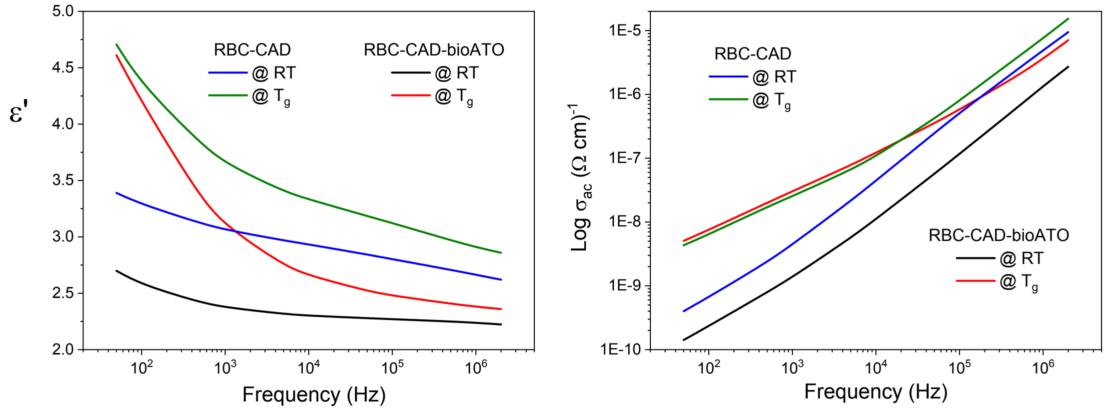

Figure S19. Dielectric permittivity and *ac* conductivity versus frequency at RT and at T_g for samples RBC-CAD and RBC-CAD-bioATO.

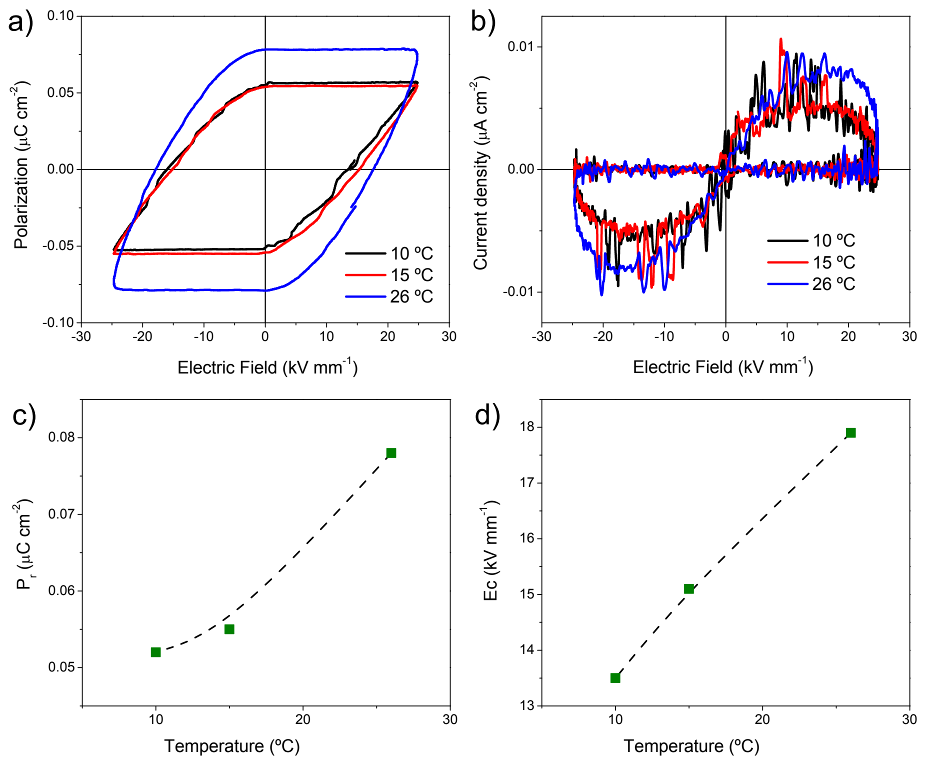

Figure S20. a) P-E hysteresis loops and b) current density curves as a function of the electric field, measured at different temperatures and 0.01 Hz, for RBC-CAD sample.

Evolution of the c) remanent polarization, P_r, and d) coercive field, E_c, as a function of temperature.

10. Computational study.

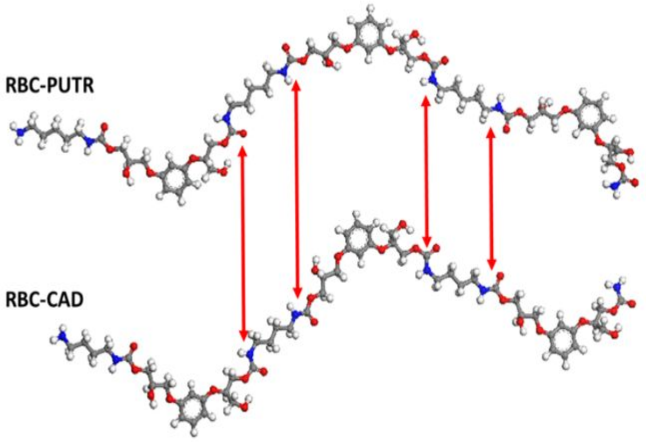

Figure S21. Comparison of the orientation of the carbonyl group in the most stable planarized structure of RBC-PUTR and RBC-CAD.

References

1. Camara, F.; Benyahya, S.; Besse, V.; Boutevin, G.; Auvergne, R.; Boutevin, B.; Caillol, S., Reactivity of secondary amines for the synthesis of non-isocyanate polyurethanes. *European Polymer Journal* 2014, *55*, 17-26.

2. Poussard, L.; Mariage, J.; Grignard, B.; Detrembleur, C.; Jérôme, C.; Calberg, C.; Heinrichs, B.; De Winter, J.; Gerbaux, P.; Raquez, J. M.; Bonnaud, L.; Dubois, P., Non-Isocyanate Polyurethanes from Carbonated Soybean Oil Using Monomeric or Oligomeric Diamines To Achieve Thermosets or Thermoplastics. *Macromolecules* 2016, *49* (6), 2162-2171.

3. Jiménez, R.; Alemany, C.; Calzada, M.; González, A.; Ricote, J.; Mendiola, J., Processing effects on the microstructure and ferroelectric properties of strontium bismuth tantalate thin films. *Applied Physics A* 2002, *75* (5), 607-615.
